# Proteins implicated in muscular dystrophy and cancer are functional constituents of the centrosome

**DOI:** 10.26508/lsa.202201367

**Published:** 2022-07-05

**Authors:** Lilli Winter, Monika Kustermann, Büsra Ernhofer, Harald Höger, Reginald E Bittner, Wolfgang M Schmidt

**Affiliations:** 1 Neuromuscular Research Department, Center for Anatomy and Cell Biology, Medical University of Vienna, Vienna, Austria; 2 Division for Laboratory Animal Science and Genetics, Medical University of Vienna, Himberg, Austria

## Abstract

Aberrant expression of dystrophin, utrophin, dysferlin, or calpain-3 was originally identified in muscular dystrophies (MDs). Increasing evidence now indicates that these proteins might act as tumor suppressors in myogenic and non-myogenic cancers. As DNA damage and somatic aneuploidy, hallmarks of cancer, are early pathological signs in MDs, we hypothesized that a common pathway might involve the centrosome. Here, we show that dystrophin, utrophin, dysferlin, and calpain-3 are functional constituents of the centrosome. In myoblasts, lack of any of these proteins caused excess centrosomes, centrosome misorientation, nuclear abnormalities, and impaired microtubule nucleation. In dystrophin double-mutants, these defects were significantly aggravated. Moreover, we demonstrate that also in non-myogenic cells, all four MD-related proteins localize to the centrosome, including the muscle-specific full-length dystrophin isoform. Therefore, MD-related proteins might share a convergent function at the centrosome in addition to their diverse, well-established muscle-specific functions. Thus, our findings support the notion that cancer-like centrosome-related defects underlie MDs and establish a novel concept linking MDs to cancer.

## Abstract (precis)

This study demonstrates that the muscular dystrophy-associated proteins dystrophin, utrophin, dysferlin, and calpain-3 localize to the centrosome and that their absence leads to excess centrosomes, compromised nuclear morphology, impaired centrosome orientation, and defective microtubule nucleation.

## Introduction

Muscular dystrophies (MDs) comprise a group of inherited disorders characterized by progressive muscle wasting and weakness. Although genetically heterogeneous, MDs share a common pathology, characterized by cycles of de- and regeneration of muscle fibers and progressive proliferation of connective tissue and fat cells. More recently, however, there is increasing evidence that aberrant expression of MD-related proteins is implicated in the development and/or progression of various types of cancer in humans and mice.

Duchenne muscular dystrophy (DMD), the most common MD of childhood, is an X-linked recessive disorder caused by mutations disrupting the dystrophin (*DMD*) gene, resulting in the absence or vast reduction of the muscle-specific full-length dystrophin protein (Dp427; Hoffman et al, 1987). Emerging evidence suggests that dystrophin also plays a role as a tumor suppressor (TS) and anti-metastatic factor (recently reviewed in Jones et al [2021]). Recently, recurrent somatic *DMD* deletions have been shown to drive development of aggressive sarcomas induced by fusion of immortalized myoblasts (Merle et al, 2020). Somatic *DMD* gene mutations and/or defective dystrophin expression has been found in various types of cancer, including non-myogenic tumors in humans (Korner et al, 2007; Wang et al, 2014; Luce et al, 2017; Gallia et al, 2018; Juratli et al, 2018; Mauduit et al, 2019). Moreover, mice lacking the expression of full-length dystrophin (*Dmd*^*mdx*^) have been found be prone to spontaneously develop age-related muscle-derived malignant sarcomas (Chamberlain et al, 2007; Fernandez et al, 2010; Schmidt et al, 2011). Although seemingly arising from skeletal muscles, these tumors presented as “mixed sarcomas”: in addition to myogenic tumor cells, also, non-myogenic compartments presenting as fibro- and liposarcomas could be identified histologically (Schmidt et al, 2011). Thus, these findings corroborate the concept that dystrophin conveys its function as TS beyond the myogenic lineage.

In addition to dystrophin, several other MD-associated proteins have been implicated in tumorigenesis (Fanzani et al, 2013). Mutations in the human *DYSF* and *CAPN3* genes are causative for autosomal recessive limb-girdle muscular dystrophy (LGMD) types R2 (Liu et al, 1998) and R1 (Richard et al, 1995). Two different *Dysf*-deficient mouse strains (SJL mutation on the C57BL/10 background and A/J) have been reported to spontaneously develop muscle-derived sarcomas, especially later in life (Schmidt et al, 2011; Sher et al, 2011). Likewise, *Capn3*-knockout mice are also susceptible to muscle-derived rhabdomyosarcomas (Schmidt et al, 2011). Significantly, the simultaneous loss of dystrophin and dysferlin (Schmidt et al, 2011; Hosur et al, 2012) or dystrophin and calpain-3 (Schmidt et al, 2011) leads to a drastically increased sarcoma propensity, which is compatible with an additive TS function of these MD-related proteins.

Utrophin, the autosomal paralogue of dystrophin, is not causatively related to MDs per se but is known to be aberrantly regulated and expressed in dystrophin-deficient conditions in humans and corresponding animal models (Helliwell et al, 1992; Matsumura et al, 1992). In addition, dystrophin-deficient *Dmd*^*mdx*^ mice additionally lacking utrophin show a dramatic aggravation of the MD phenotype, leading to premature death, suggesting a cooperative interaction of both molecules (Grady et al, 1997; Deconinck et al, 1997b). Notably, it is also proposed as a TS candidate as the *UTRN* gene has been found to be mutated in human cancers, like breast cancers, neuroblastomas, and malignant melanomas and as overexpression of utrophin in breast cancer cells inhibits growth (Li et al, 2007).

The fact that each of the four proteins described above, dystrophin, utrophin, dysferlin, and calpain-3, are implicated in the pathobiology of hitherto unrelated conditions, that is, MDs and cancer, tempted us to speculate that there must be a unifying “player” on the cellular level. However, the functional basis underlying the proposed TS properties of MD-related proteins remained elusive so far. Because dysfunctional centrosomes are causatively involved in DNA damage and somatic aneuploidy, both of which are hallmarks of cancer and in MDs, where these changes are present in muscles already before dystrophic changes, we set out to directly address this hypothesis experimentally.

## Results

### Dystrophin is a constituent of the centrosome in myoblasts and non-myogenic cells

**Figure 1.**
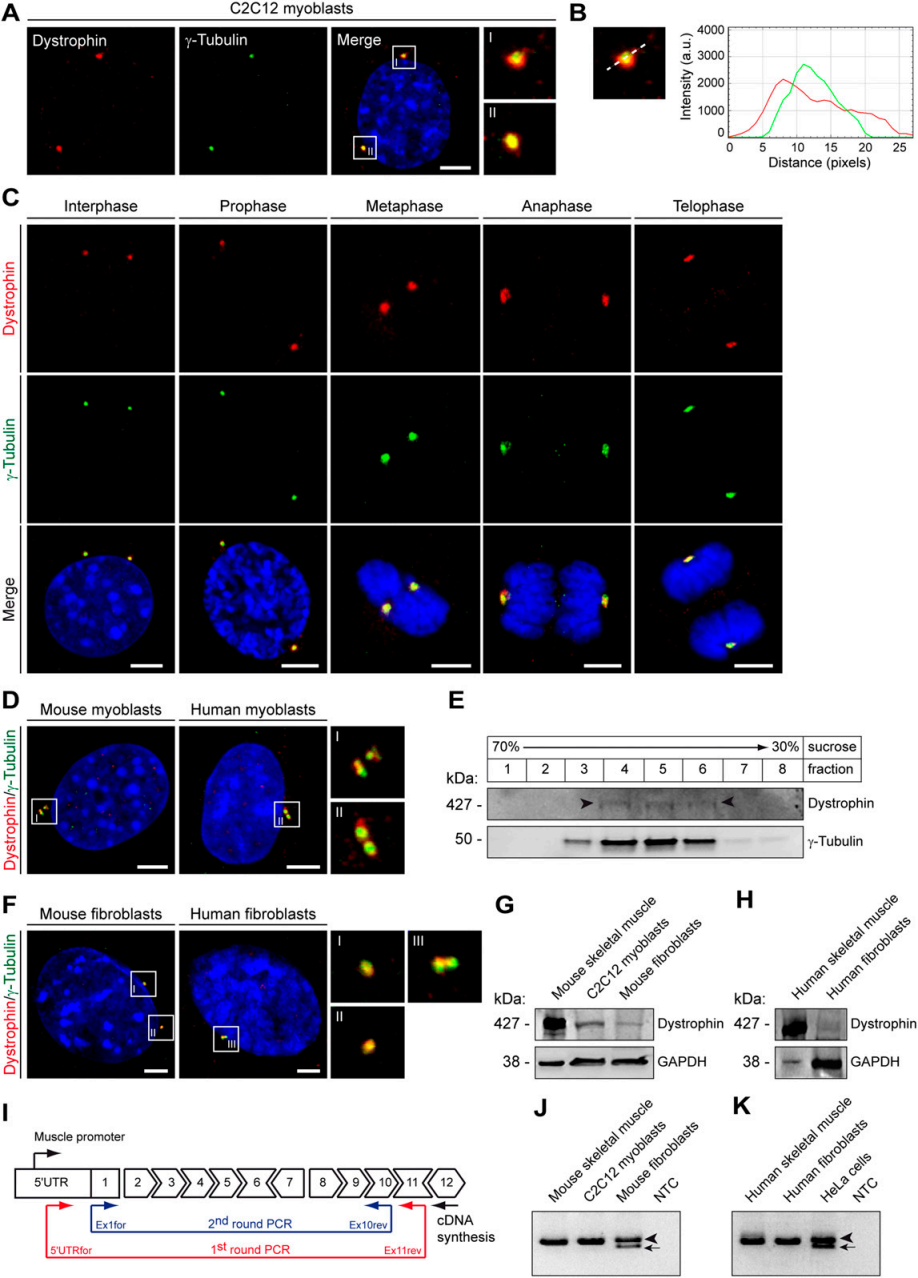
Dystrophin localizes to the centrosome. **(A)** Double immunocytochemistry (ICC) of C2C12 myoblasts using antibodies specific for dystrophin (red) and γ-tubulin (green) and visualization of nuclei (DAPI, blue). Insets are magnifications of the centrosomes (as indicated by the boxes I and II). Note the co-localization of dystrophin with γ-tubulin. **(B)** Fluorescence intensity plots illustrating the respective distribution of dystrophin (red) and γ-tubulin (green). The white dashed line denotes the direction of the profiling through the centrosome (shown in inset I in A). Note that dystrophin overlaps the γ-tubulin-positive area. a.u., arbitrary units. **(A, C)** ICC of proliferating C2C12 myoblasts as in (A) at different phases of the cell cycle. **(A, D)** ICC of primary murine and human myoblasts as described in **(A)**. **(E)** Co-purification of dystrophin with γ-tubulin in centrosome-enriched subcellular fractions. Centrosomes were purified from C2C12 myoblasts by sucrose density-gradient centrifugation. Fractions were analyzed by immunoblotting. Note that in centrosome-enriched fractions 4, 5, and 6, as evaluated by increased γ-tubulin protein levels, also full-length dystrophin (arrowheads) was detected. **(F)** ICC of murine (*p53*^*−/−*^) and human (WI-38) fibroblasts using antibodies specific for dystrophin (red) and γ-tubulin (green) and visualization of nuclei (DAPI, blue). Insets are magnifications of the centrosomes (as indicated by the boxes I, II, and III). Scale bars **(A, C, D, F)**, 5 μm. **(G)** Expression of dystrophin in murine skeletal-muscle lysates and cell lysates prepared from mouse myoblasts (C2C12) and fibroblasts (*p53*^*−/−*^). **(H)** Expression of dystrophin in human skeletal-muscle lysate and cell lysate prepared from human fibroblasts (WI-38). GAPDH **(G, H)**, loading control. **(I)** Scheme illustrating the nested RT–PCR strategy used to detect dystrophin transcripts starting from the dystrophin muscle promoter (muscle-specific exon 1) extending into exon 11. As full-length dystrophin (Dp427) isoforms start from individual promoters (cortical/brain, muscle, or Purkinje cell promoter) into unique first exons followed by a common exon 2 (Muntoni et al, 2003; Doorenweerd et al, 2017); this strategy enabled the specific detection of dystrophin transcripts expressed from the muscle-specific promoter only. Moreover, as shorter dystrophin isoforms usually lack the amino-terminal dystrophin amino acids, RT–PCR products obtained by this strategy are likely to correspond to full-length Dp427m. **(J)** Representative results of nested RT–PCR for mouse dystrophin. Note that for mouse fibroblasts, in addition to the expected 1,113-bp PCR product (arrowhead), a second band of 981 bp was observed (arrow), corresponding to a transcript lacking exon 9 (Reiss & Rininsland, 1994). **(K)** Representative results of nested RT–PCR for human dystrophin. Note that for HeLa cells, in addition to the expected 1,024-bp PCR product (arrowhead), a second band of 895 bp was observed (arrow), corresponding to skipping of exon 9 (Reiss & Rininsland, 1994) in this cell line as well. NTC (J, K), no template control. The specificity of all PCR products in J and K was verified by DNA sequencing. Source data are available for this figure.

**Figure S1.**
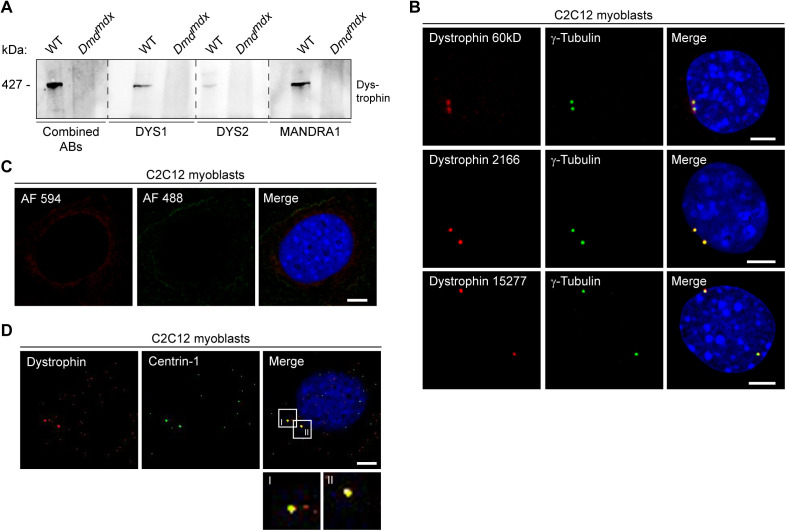
Enhancement of dystrophin detection by usage of combined antibodies, co-localization of dystrophin and centrosomes (γ-tubulin or centrin-1) in C2C12 cells, and corresponding negative controls. **(A)** Equal amounts of skeletal-muscle lysates obtained from wild-type (WT) and *Dmd*^*mdx*^ mice were subjected to immunoblotting using dystrophin-specific antibodies DYS1, DYS2, and MANDRA1 or a combination of all three dystrophin antibodies (combined ABs). Note the increased intensity of the dystrophin-specific band in the lane where combined antibodies were used. **(B)** Immunocytochemistry (ICC) of proliferating C2C12 myoblasts using polyclonal antisera specific for dystrophin (sheep antiserum 60 kD, rabbit antiserum 2166, or rabbit antiserum 15,277, all in red) and γ-tubulin (green); nuclei were visualized with DAPI (blue). **(C)** Secondary antibodies only (Alexa Fluor (AF) 594 or AF 488) were used for negative controls. **(D)** ICC of proliferating C2C12 myoblasts using antibodies specific for dystrophin (red) and centrin-1 (green); nuclei were visualized with DAPI (blue). Scale bars (B, C, D), 5 μm. Source data are available for this figure.

**Figure S2.**
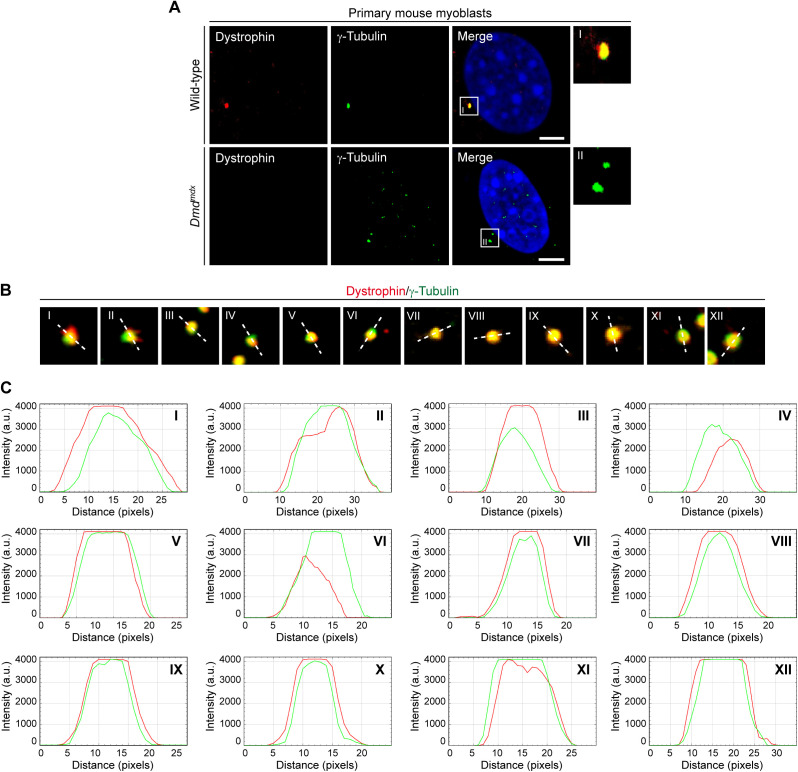
Co-localization of dystrophin and γ-tubulin in WT, but not in *Dmd*^*mdx*^ myoblasts, and evaluation of the spatial centrosomal localization of dystrophin. **(A)** ICC of primary wild-type and *Dmd*^*mdx*^ myoblasts using antibodies specific for dystrophin (red) and γ-tubulin (green); nuclei were visualized with DAPI (blue). Note that no dystrophin signal was observed in *Dmd*^*mdx*^ myoblasts. Scale bar, 5 μm. **(B)** Representative ICC of dystrophin (red) and γ-tubulin (green) showing 12 different centrosomes from C2C12 myoblasts. The white dashed line denotes the direction of the profiling through the centrosome. **(B, C)** Fluorescence intensity plots illustrating the respective distribution of dystrophin (red) and γ-tubulin (green) from centrosomes shown in (B). a.u., arbitrary units.

To test the hypothesis that dystrophin might play a role at the centrosome, we first investigated its subcellular localization in proliferating C2C12 myoblasts by immunocytochemistry (ICC). Most frequently, we observed two dot-like signals mostly located in close vicinity to the cell nucleus, thus reminiscent of centrosomes. We confirmed this by co-probing the myoblasts for two centrosomal marker proteins, which resulted in staining patterns characterized by widely overlapping immunosignals corresponding to dystrophin and γ-tubulin (Figs 1A and B and S1A and B and Video 1; secondary antibody controls for ICC experiments are shown in Fig S1C) or centrin-1 (Fig S1D). Although several antibodies rose against different epitopes of dystrophin that stained the centrosome (Fig S1A and B), no dystrophin signals were obtained in myoblasts derived from *Dmd*^*mdx*^ mice (Fig S2A). Evaluation of the spatial centrosomal organization of dystrophin revealed a clear co-distribution with γ-tubulin (Figs 1B and S2B and C). Moreover, we found that centrosomal localization of dystrophin was maintained throughout the entire cell division cycle (Fig 1C). Thus, our findings established co-localization of dystrophin and the centrosome in proliferating C2C12 myoblasts. In a next step, we co-stained murine and human primary myoblasts for dystrophin and γ-tubulin, respectively, and again detected a co-localization of these proteins at the centrosome (Fig 1D).

Video 1 Animated projection of the dystrophin localization at the centrosome. 3D projection of a C2C12 myoblast immunolabeled for dystrophin (red) and γ-tubulin (green) and visualization of nuclei (DAPI, blue), as shown in Fig 1A. Download video

To address the question if dystrophin is a constituent of the centrosomal multi-protein complex, we isolated centrosomes from C2C12 myoblasts by density-gradient centrifugation. Probing the centrosome-enriched fractions for dystrophin expression by Western blotting (WB) gave rise to an immunoreactive band at the position of the full-length skeletal-muscle isoform Dp427m (Fig 1E) (Hoffman et al, 1987).

**Figure S3.**
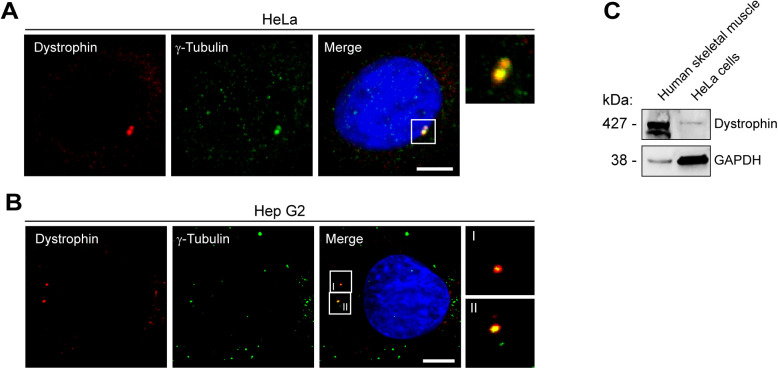
Expression and centrosomal localization of dystrophin in HeLa and Hep G2 cells. **(A)** ICC of HeLa cells using antibodies specific for dystrophin (DYS3, red) and γ-tubulin (green) and visualization of nuclei (DAPI, blue). Inset is a magnification of the boxed centrosomes. Note the co-localization of dystrophin with γ-tubulin. Scale bar, 5 μm. **(B)** ICC of Hep G2 cells using antibodies specific for dystrophin (combined DYS2 and MANDRA1, red) and γ-tubulin (green) and visualization of nuclei (DAPI, blue). Insets show magnifications of the boxed centrosomes (as indicated by I-II). Note the co-localization of dystrophin with γ-tubulin. Scale bar, 5 μm. **(C)** Expression of dystrophin (using a combination of DYS1, DYS2, and MANDRA1 antibodies) in human skeletal-muscle lysates and cell lysates prepared from HeLa cells. GAPDH, loading control. Source data are available for this figure.

Because defective dystrophin expression has been implicated not only in myogenic but also non-myogenic cancers in mice and men (Schmidt et al, 2011; Wang et al, 2014), we hypothesized that the canonical full-length dystrophin isoform Dp427m might be a centrosome constituent also in cells other than muscle cells. Therefore, we probed murine (*p53*^*−/−*^) dermal and human (WI-38) lung fibroblasts as well as human carcinoma cells (HeLa, Hep G2) for dystrophin and γ-tubulin expression. Also, in these cells, we observed a co-localization of respective immunosignals (Figs 1F and S3A and B). Using highly concentrated protein extracts from murine and human fibroblasts as well as from HeLa cells, we could detect a dystrophin-specific immunoreactive band by WB at 427 kDa (Figs 1G and H and S3C). To verify that Dp427m is a centrosomal constituent in non-muscle cells, we tested these cells for the expression of the canonical muscle promoter of dystrophin and indeed found it to be expressed on the RNA level (Fig 1I–K). Taken together, our experiments demonstrate that the Dp427m is a centrosomal protein in myoblasts and also in cells not belonging to the myogenic lineage.

### Utrophin, dysferlin, and calpain-3 also localize to the centrosome in myoblasts and fibroblasts

**Figure 2.**
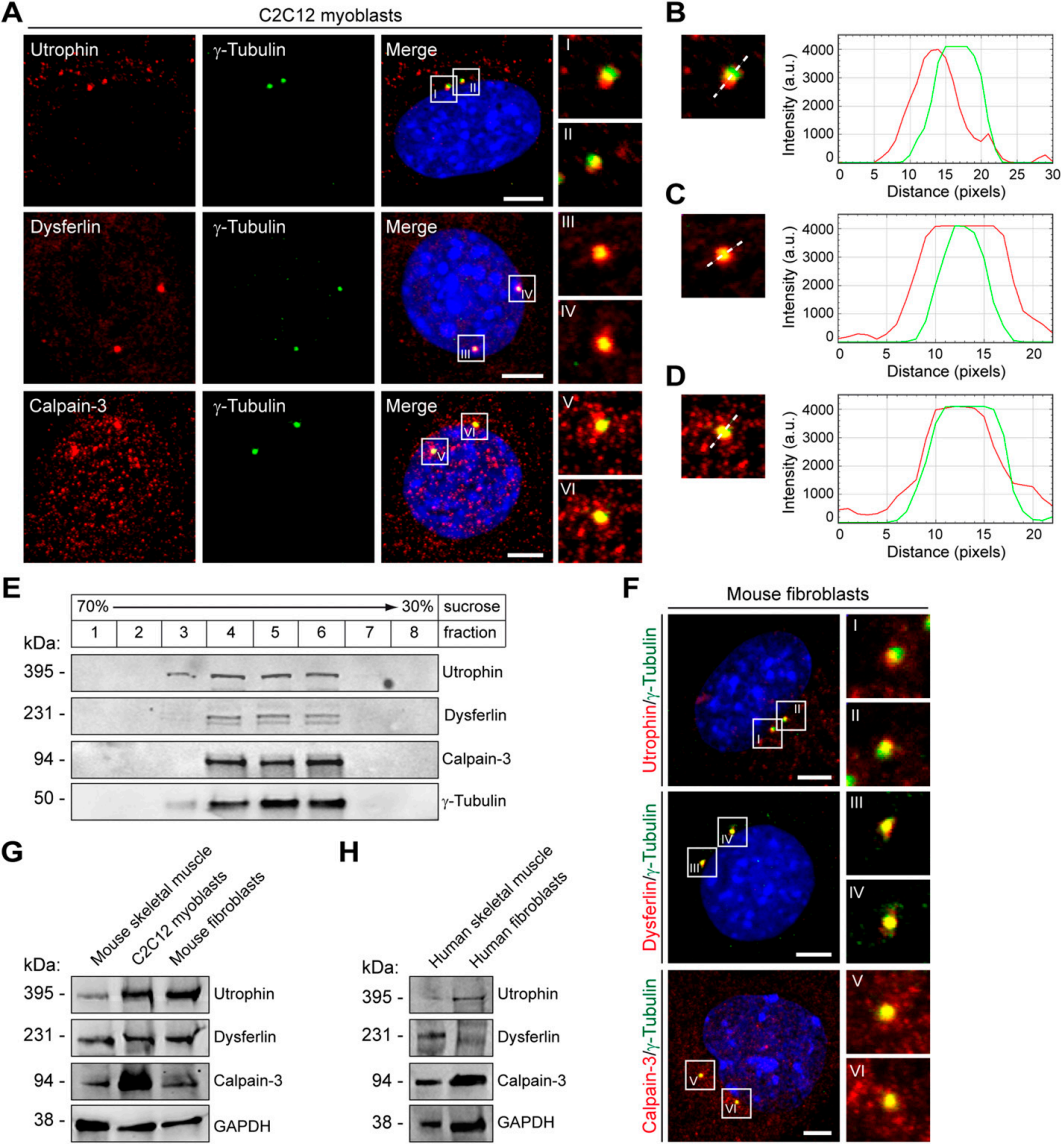
Utrophin, dysferlin, and calpain-3 also localize to the centrosome. **(A)** ICC of proliferating C2C12 cells using antibodies specific for utrophin, dysferlin, and calpain-3, respectively (all in red), in combination with antibodies specific for γ-tubulin (green), and visualization of nuclei (DAPI, blue). Insets are magnifications of the boxed centrosomes (as indicated by I–VI). Note the co-localization of muscular dystrophy–related proteins with γ-tubulin. Scale bars, 5 μm. **(B, C, D)** Fluorescence intensity plots illustrating the distributions of utrophin (B), dysferlin (C), and calpain-3 (D), respectively (all in red) and γ-tubulin (green). **(A)** The white dashed lines denote the direction of the profiling through the respective centrosome (shown in insets I, III, and VI in A). Note that utrophin, dysferlin, and calpain-3 overlap the respective γ-tubulin-positive area. a.u., arbitrary units. **(E)** Co-purification of utrophin, dysferlin, and calpain-3 with γ-tubulin. Centrosomes were purified from C2C12 myoblasts by sucrose density-gradient centrifugation. Fractions were analyzed by immunoblotting. Note that in centrosome-enriched fractions 4, 5, and 6, as evaluated by increased γ-tubulin protein levels, also full-length utrophin, dysferlin, and calpain-3 were detected. **(A, F)** ICC of mouse fibroblasts (*p53*^*−/−*^) as described in (A). Insets are magnifications of the boxed centrosomes (I–VI). Scale bars, 5 μm. **(G)** Expression of utrophin, dysferlin, and calpain-3 in murine skeletal-muscle lysates and cell lysates prepared from mouse myoblasts (C2C12) and fibroblasts (*p53*^*−/−*^). **(H)** Expression of utrophin, dysferlin, and calpain-3 in human skeletal-muscle lysate and cell lysate prepared from human fibroblasts (WI-38). GAPDH (G, H), loading control. Source data are available for this figure.

**Figure S4.**
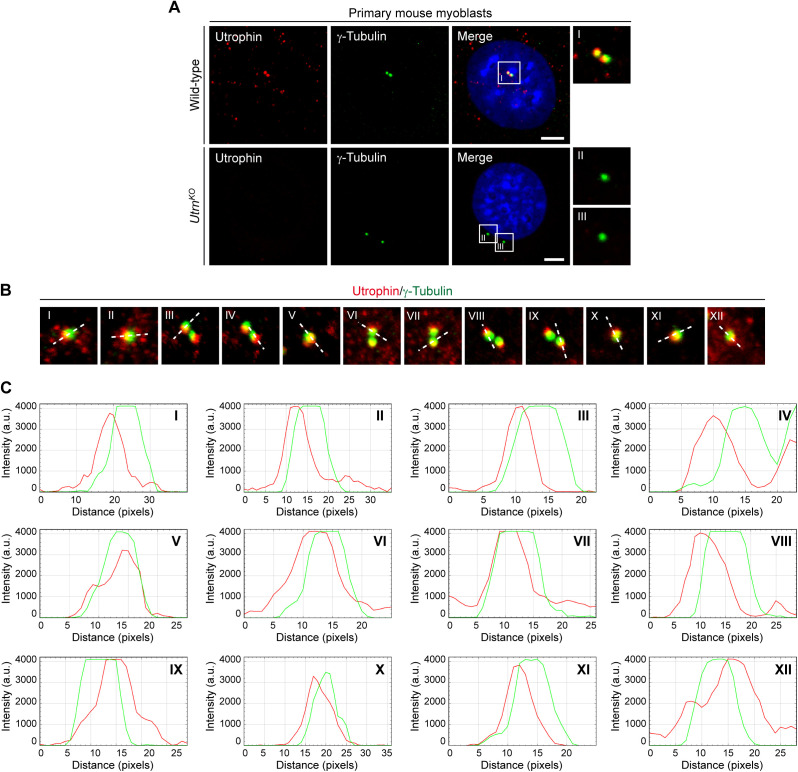
Co-localization of utrophin and γ-tubulin in WT, but not in *Utrn^KO^* myoblasts, and evaluation of the spatial centrosomal localization of utrophin. **(A)** ICC of primary wild-type and *Utrn*^*KO*^ myoblasts using antibodies specific for utrophin (red) and γ-tubulin (green); nuclei were visualized with DAPI (blue). Note that no utrophin signal was observed in *Utrn*^*KO*^ myoblasts. Scale bar, 5 μm. **(B)** Representative ICC of utrophin (red) and γ-tubulin (green) showing 12 different centrosomes from C2C12 myoblasts. The white dashed line denotes the direction of the profiling through the centrosome. **(B, C)** Fluorescence intensity plots illustrating the respective distribution of utrophin (red) and γ-tubulin (green) from centrosomes shown in (B). a.u., arbitrary units.

**Figure S5.**
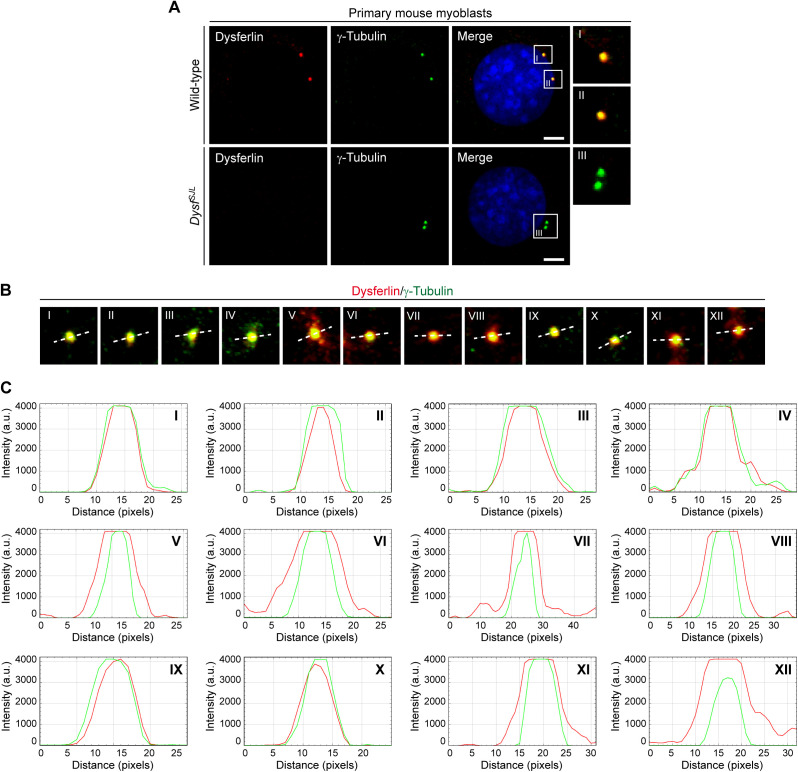
Co-localization of dysferlin and γ-tubulin in WT, but not in *Dysf*^*SJL*^ myoblasts, and evaluation of the spatial centrosomal localization of dysferlin. **(A)** ICC of primary wild-type and *Dysf*^*SJL*^ myoblasts using antibodies specific for dysferlin (red) and γ-tubulin (green); nuclei were visualized with DAPI (blue). Note that no dysferlin signal was observed in *Dysf*^*SJL*^ myoblasts. Scale bar, 5 μm. **(B)** Representative ICC of dysferlin (red) and γ-tubulin (green) showing 12 different centrosomes from C2C12 myoblasts. The white dashed line denotes the direction of the profiling through the centrosome. **(B, C)** Fluorescence intensity plots illustrating the respective distribution of dysferlin (red) and γ-tubulin (green) from centrosomes shown in (B). a.u., arbitrary units.

**Figure S6.**
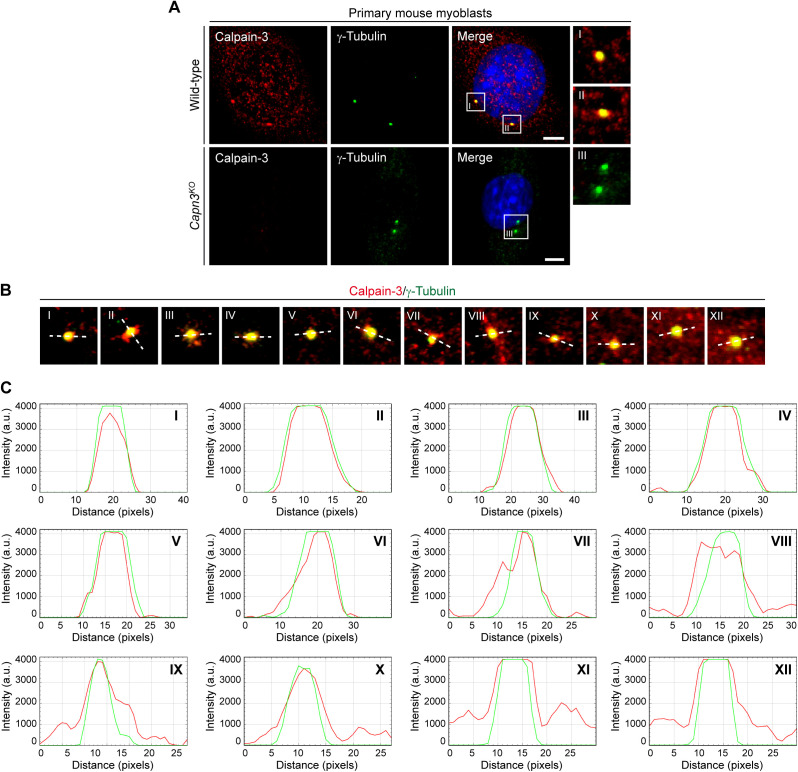
Co-localization of calpain-3 and γ-tubulin in WT, but not in *Capn3*^*KO*^ myoblasts, and evaluation of the spatial centrosomal localization of calpain-3. **(A)** ICC of primary wild-type and *Capn3*^*KO*^ myoblasts using antibodies specific for calpain-3 (red) and γ-tubulin (green); nuclei were visualized with DAPI (blue). Note that no calpain-3 signal was observed in *Capn3*^*KO*^ myoblasts. Scale bar, 5 μm. **(B)** Representative ICC of calpain-3 (red) and γ-tubulin (green) showing 12 different centrosomes from C2C12 myoblasts. The white dashed line denotes the direction of the profiling through the centrosome. **(B, C)** Fluorescence intensity plots illustrating the respective distribution of calpain-3 (red) and γ-tubulin (green) from centrosomes shown in (B). a.u., arbitrary units.

**Figure S7.**
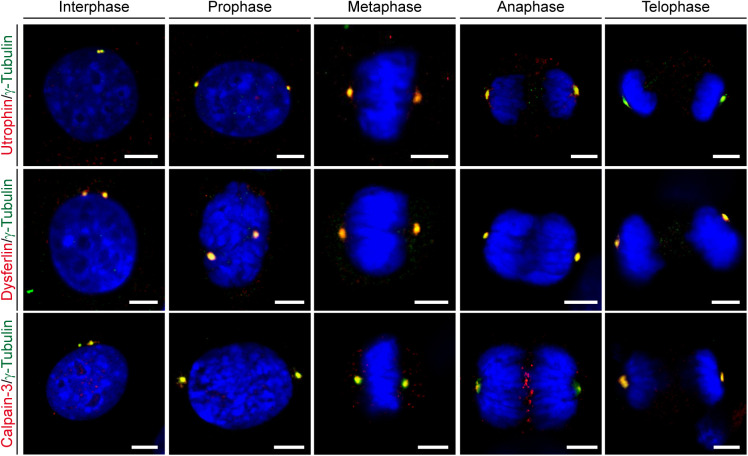
Utrophin, dysferlin, and calpain-3 localize to the centrosome in different phases of the cell cycle. ICC of C2C12 myoblasts using antibodies specific for utrophin, dysferlin, and calpain-3, respectively (all in red), in combination with antibodies specific for γ-tubulin (green) and visualization of nuclei (DAPI, blue). Note that all three proteins co-localize with γ-tubulin in all phases of the cell cycle shown. Scale bars, 5 μm.

Next, we examined whether other MD-related proteins are also constituents of the centrosome. To this end, we performed ICC in C2C12 myoblasts to probe also for the subcellular localization of utrophin, dysferlin, and calpain-3, respectively. As for dystrophin, we consistently found a close association of dot-like immunosignals with γ-tubulin for all three MD-related proteins in C2C12 cells (Fig 2A–D and Video 2–Video 4) and primary mouse myoblasts but not in myoblasts derived from mutant *Utrn*^*KO*^, *Dysf*^*SJL*^, or *Capn3*^*KO*^ mice, respectively (Figs S4A–C, S5A–C, and S6A–C). Moreover, also, these three MD proteins were localized to the centrosome throughout the entire cell division cycle (Fig S7).

Video 2 Animated projection of the utrophin localization at the centrosome. 3D projection of a C2C12 myoblast immunolabeled for utrophin (red) and γ-tubulin (green) and visualization of nuclei (DAPI, blue), as shown in Fig 2A. Download video

Video 3 Animated projection of the dysferlin localization at the centrosome. 3D projection of a C2C12 myoblast immunolabeled for dysferlin (red) and γ-tubulin (green) and visualization of nuclei (DAPI, blue), as shown in Fig 2A. Download video

Video 4 Animated projection of the calpain-3 localization at the centrosome. 3D projection of a C2C12 myoblast immunolabeled for calpain-3 (red) and γ-tubulin (green) and visualization of nuclei (DAPI, blue), as shown in Fig 2A. Download video

To test if these proteins are also biochemically linked to the centrosome complex, we probed centrosome-enriched cellular fractions by WB. Antibodies against utrophin, dysferlin, and calpain-3, respectively, gave rise to bands of apparent molecular weights, which were indicative for the full-length isoforms of the respective proteins (Fig 2E). The fact that defective expression of these proteins has been found in cancers of different tissue types prompted us to speculate that centrosomal expression of these proteins might not be confined to myogenic cells. Therefore, we next probed murine fibroblasts by double ICC for the respective proteins with γ-tubulin and observed a centrosome-associated localization of utrophin, dysferlin, and calpain-3 (Fig 2F). Moreover, when we tested protein lysates prepared from murine and human fibroblasts by WB, we confirmed high-level expression of canonical full-length isoforms also in these cell types (Fig 2G and H).

**Figure S8.**
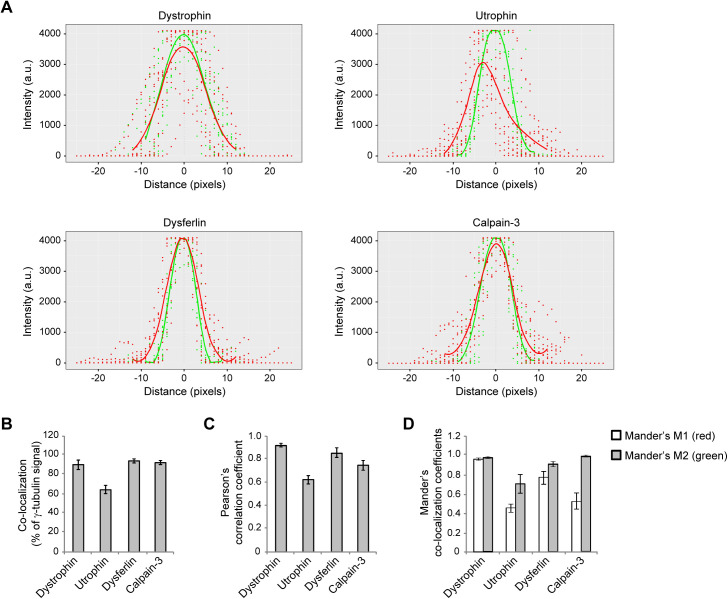
Co-localization analyses of muscular dystrophy (MD) proteins and γ-tubulin. **(A)** Plot of fluorescence intensity signals as shown in Figs S2C, S4C, S5C, and S6C, illustrating the distributions of dystrophin, utrophin, dysferlin, and calpain-3 (red), with respect to γ-tubulin (green). For easier comparison, data from all four analyses were centered around the maximum of the corresponding γ-tubulin signal, keeping 25 pixels in either direction. Note that although dystrophin, dysferlin, and calpain-3 are closely overlapping with γ-tubulin, utrophin appears more distally associated with the centrosome. Data are shown as overplots of all points from individual maximum projections; lines represent smoothed conditional means (calculated from local polynomial regression, using the method “stats::loess” in R) (n = 12 centrosomes per MD protein). **(B)** Statistical evaluation of the area covered by overlapping dystrophin, utrophin, dysferlin, and calpain-3 signals with γ-tubulin signals in the fluorescence intensity plots depicted in S2C, S4C, S5C, and S6C in relation to the total γ-tubulin signals. Mean ± SEM (n = 12 centrosomes per MD protein). **(C)** Bar graphs show the Pearson’s correlation coefficients for the overlay of MD protein-specific fluorescence signals with γ-tubulin. Note that Pearson’s correlation coefficients, a measure of linear correlation, indicate for all four MD proteins a high probability that pixels of both channels are overlay. **(D)** Statistical evaluation of the Mander’s co-localization coefficients, well-established co-occurrence measures that calculate the percentage of total signal from one channel which overlaps with the signal from the other channel (Manders et al, 1993). Mander’s M1 value, fraction of the respective MD protein (red channel) co-localized with γ-tubulin (green channel). Mander’s M2 value, fraction of γ-tubulin (green channel) co-localized with the respective MD protein (red channel). Note that although dystrophin and dysferlin co-localized with γ-tubulin to 95% and 76% (M1 value, red signal), respectively, and 97% and 91% of the γ-tubulin overlapped with the corresponding dystrophin and dysferlin pixels (M2 value, green signal), respectively; the analysis for utrophin revealed a M1 value of 0.45 and a M2 value of 0.7, indicating the several pixels of each color were not co-localizing with each other. For calpain-3, the M1 value was 0.5, whereas the M2 value was 0.99, indicating that several red (calpain-3 positive, cytoplasmic) signals that were not overlapping with green signals, whereas almost all γ–tubulin-positive signals were also positive for calpain-3. Mean ± SEM (n = 12 centrosomes per MD protein).

Even though all four MD proteins displayed close spatial association of dot-like immunosignals with γ-tubulin in C2C12 cells, utrophin seemed to be more distally shifted, whereas dystrophin, dysferlin, and calpain-3 appeared to more clearly overlap with the maximum of centrosomal γ-tubulin signal (Fig S8A). Likewise, statistical evaluation of the co-localization with γ-tubulin-positive signals revealed ∼90% overlap with dystrophin, dysferlin, and calpain-3 but only ∼65% with utrophin (Fig S8B). In addition, the evaluation of the Pearson’s and Mander’s coefficients indicated that all four MD-related proteins studied herein clearly co-localized with the centrosome (Fig S8C and D).

### Centrosome amplification in human and murine MD myoblasts

**Figure 3.**
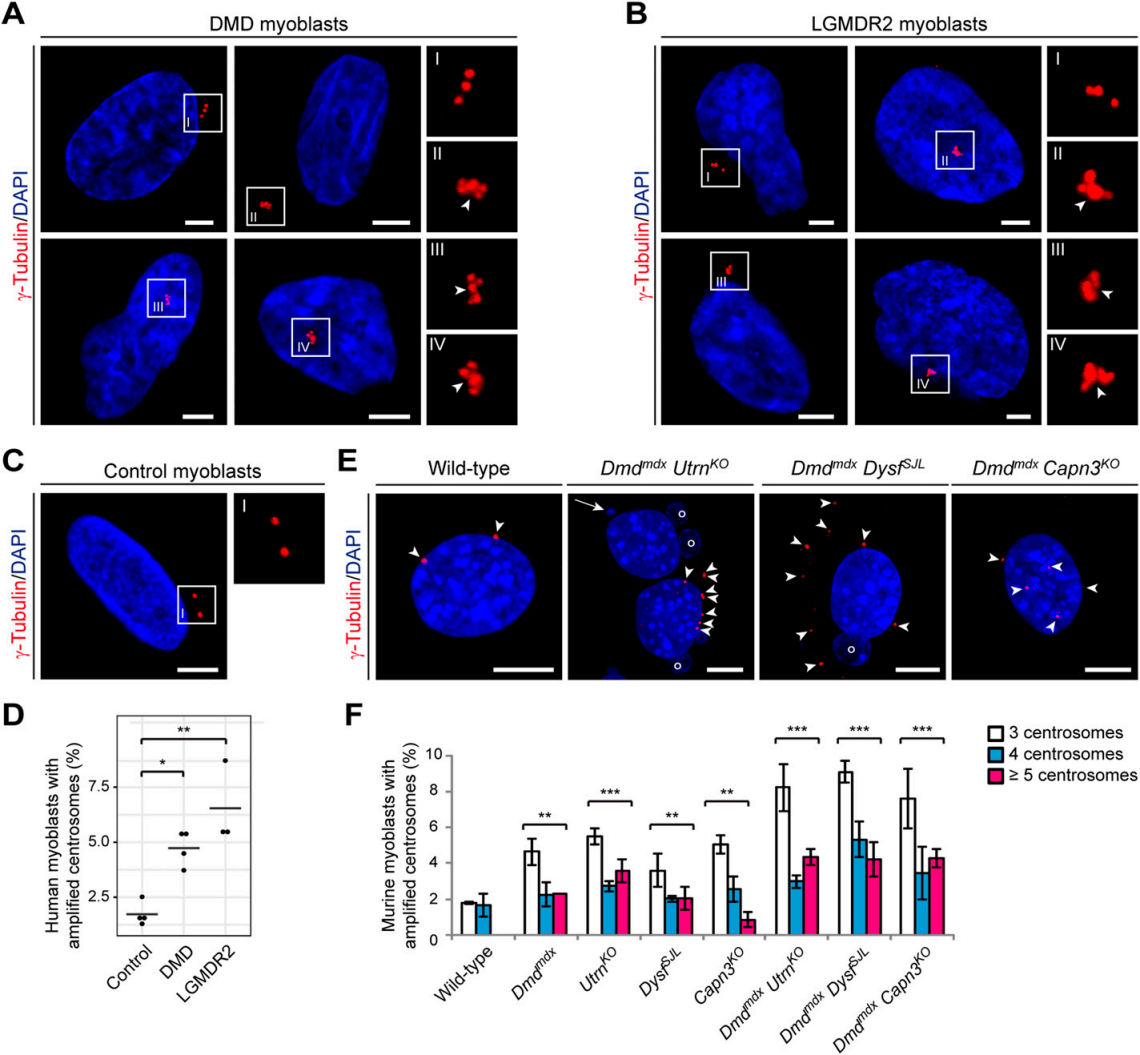
Centrosome amplification in human and murine muscular dystrophy (MD) myoblasts. **(A, B, C)** Representative ICC of centrosomes (γ-tubulin, red) and nuclei (DAPI) in human myoblasts derived from Duchenne muscular dystrophy (DMD) (A) or LGMDR2 (B) patients or healthy controls (C). Insets are magnifications of the boxed centrosomes. **(A, B)** Arrowheads in (A, I–IV) and (B, I–IV) indicate clustered centrosomes. Scale bars, 5 μm. **(D)** Statistical analyses of the percentage of control, DMD, or LGMDR2 myoblasts harboring ≥3 centrosomes. Data presented as values (dots) and median (line); (control [n = 4; analyses were performed on duplicates of two controls], DMD [n = 4], LGMDR2 [n = 3]). *P < 0.05, **P < 0.01 (one-way ANOVA and post hoc Tukey correction). **(E)** ICC of wild-type (WT) and MD double-mutant murine myoblasts using γ-tubulin antibody (red) and visualization of nuclei (DAPI, blue). Arrowheads indicate centrosomes. Note that double-mutant MD myoblasts display supernumerary centrosomes. Also note the occurrence of nuclear abnormalities such as micronuclei (long arrow) and blebs (circles) in *Dmd*^*mdx*^ *Utrn*^*KO*^ and *Dmd*^*mdx*^ *Dysf*^*SJL*^ myoblasts. Moreover, note that a binucleated myoblast is shown for *Dmd*^*mdx*^ *Utrn*^*KO*^. Scale bars, 5 μm. **(F)** Percentage of murine myoblasts harboring 3 (white bars), 4 (blue bars), or ≥5 centrosomes (magenta bars) per cell. Mean ± SEM (WT [n = 320], *Dmd*^*mdx*^ [n = 306], *Utrn*^*KO*^ [n = 324], *Dysf*^*SJL*^ [n = 303], *Capn3*^*KO*^ [n = 316], *Dmd*^*mdx*^ *Utrn*^*KO*^ [n = 310], *Dmd*^*mdx*^ *Dysf*^*SJL*^ [n = 336], *Dmd*^*mdx*^ *Capn3*^*KO*^ [n = 319 myoblasts], cells were isolated from three to four newborn mice each). **P < 0.01, ***P < 0.001 (Fisher’s exact test, compared with WT myoblasts; cells with normal centrosome counts (1–2) versus cells with supernumerary centrosomes).

Chromosomal instability and aneuploidy, which we have previously shown to affect skeletal muscle and cultured myoblasts from human MD patients (Schmidt et al, 2011), are known to be causatively related to centrosome amplification (Cosenza & Kramer, 2016). Thus, we next determined the numbers of centrosomes in primary myoblasts derived from MD patients. We detected significantly increased numbers of cells containing amplified centrosomes (∼5% of DMD and ∼7% of LGMDR2 myoblasts, respectively), as compared with less than 2% of myoblasts derived from healthy (control) individuals (Fig 3A–D). Supernumerary centrosomes, as found in myoblasts derived from MD patients, often displayed immunosignals, which were suggestive for centrosome clustering (Fig 3A and B, see insets II-IV). Centrosome amplification was also found to occur at a significantly higher level in primary murine myoblasts derived from neonatal single-mutant (*Dmd*^*mdx*^, *Utrn*^*KO*^, *Dysf*^*SJL*^, *Capn3*^*KO*^) MD mice as compared with wild type (WT). In myoblasts from double-mutant (*Dmd*^*mdx*^ *Utrn*^*KO*^, *Dmd*^*mdx*^ *Capn3*^*KO*^, *Dmd*^*mdx*^ *Dysf*^*SJL*^) mice, centrosome amplification was even more increased, highly suggestive of a negative additive effect (Fig 3E and F).

### Abnormal nuclear morphology in murine and human MD myoblasts

**Figure 4.**
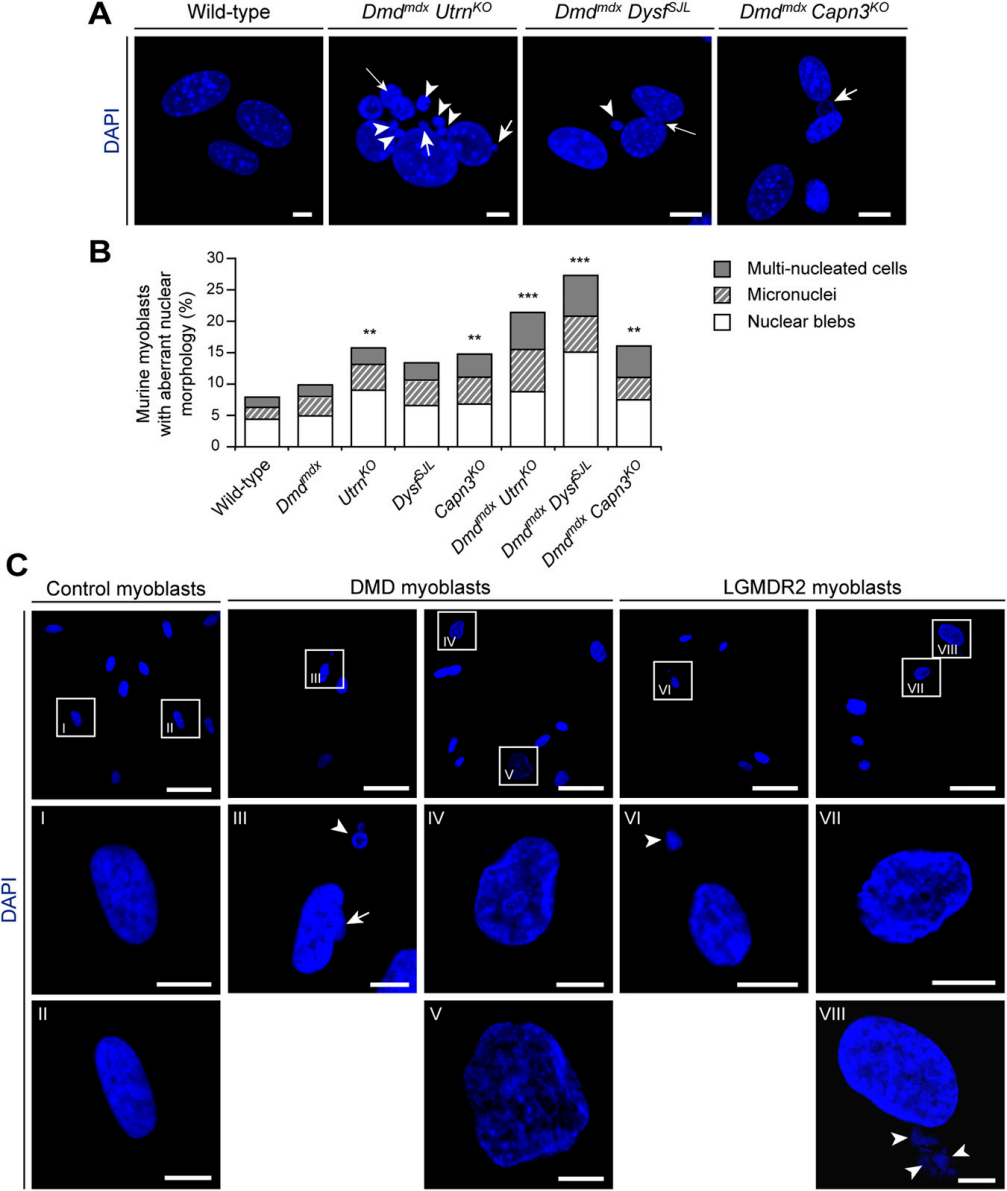
Altered nuclear morphology in murine and human muscular dystrophy myoblasts. **(A)** Visualization of nuclear morphology (DAPI) in WT and double-mutant primary myoblasts. Note the occurrence of nuclear abnormalities such as blebs (short arrows) and micronuclei (arrowheads) in double-mutant muscular dystrophy myoblasts. Also note the presence of multiple nuclei within a single myoblast (long thin arrows). Scale bars, 5 μm. **(A, B)** Statistical evaluation of primary myoblasts harboring nuclear abnormalities as depicted in (A). (WT [n = 320], *Dmd*^*mdx*^ [n = 306], *Utrn*^*KO*^ [n = 324], *Dysf*^*SJL*^ [n = 303], *Capn3*^*KO*^ [n = 316], *Dmd*^*mdx*^ *Utrn*^*KO*^ [n = 310], *Dmd*^*mdx*^ *Dysf*^*SJL*^ [n = 336], *Dmd*^*mdx*^ *Capn3*^*KO*^ [n = 319 myoblasts], cells were isolated from three to four newborn mice each). **(C)** Visualization of nuclear morphology (DAPI) in primary myoblasts derived from control samples and Duchenne muscular dystrophy or LGMDR2 patients. Lower panels (I–VIII) are magnifications of the boxed nuclei in the upper panel. Note the occurrence of nuclear abnormalities such as altered shape and reduced roundness, giant nuclei (IV, V, VII, VIII), blebs (arrow), or micronuclei (arrowheads) in patient-derived cells. Scale bars, 50 μm (upper panel), 10 μm (lower panels). **P < 0.01, ***P < 0.001 (Fisher’s exact test, compared with WT myoblasts; cells with normal nuclei versus cells with altered nuclear morphology).

**Figure S9.**
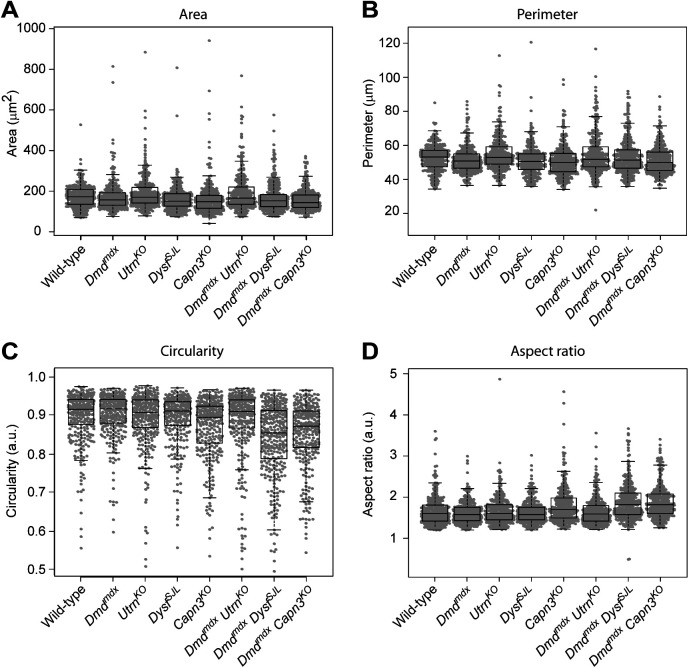
Evaluation of nuclear parameters in myoblasts derived from single- or double-mutant muscular dystrophy mice. **(A, B, C, D)** Box plots showing area (A), perimeter (B), circularity (C), and aspect ratio (D). Data are represented by “beeswarm” (gray points) box plots (black) (wild-type [n = 320], *Dmd*^*mdx*^ [n = 306], *Utrn*^*KO*^ [n = 324], *Dysf*^*SJL*^ [n = 303], *Capn3*^*KO*^ [n = 316], *Dmd*^*mdx*^ *Utrn*^*KO*^ [n = 310], *Dmd*^*mdx*^ *Dysf*^*SJL*^ [n = 336], *Dmd*^*mdx*^ *Capn3*^*KO*^ [n = 319 myoblasts], cells were isolated from three to four newborn mice each). a.u., arbitrary units.

Because centrosome amplification also impairs the integrity of the cell nucleus (Pihan, 2013), we counted nuclei displaying abnormal morphologies (i.e., occurrence of micronuclei, nuclear blebs, or multinucleated cells) in murine single- and double-mutant MD myoblasts. We detected a significantly increased number of nuclei displaying abnormal morphologies in *Utrn*^*KO*^ and *Capn3*^*KO*^ myoblasts as compared with WT cells. In all double-mutant MD myoblasts, these nuclear pathologies were even more pronounced (Fig 4A and B), whereas the analyses of other morphometric parameters of nuclei such as area, perimeter, circularity, or aspect ratio revealed no statistically significant differences (Fig S9A–D). Likewise, we could frequently detect pronounced nuclear abnormalities such as altered shapes and reduced roundness, giant nuclei, nuclear blebbing, or micronuclei in human myoblasts derived from patients affected by DMD or LGMDR2, respectively (Fig 4C).

### Impaired centrosome reorientation and microtubule regrowth in MD myoblasts

**Figure 5.**
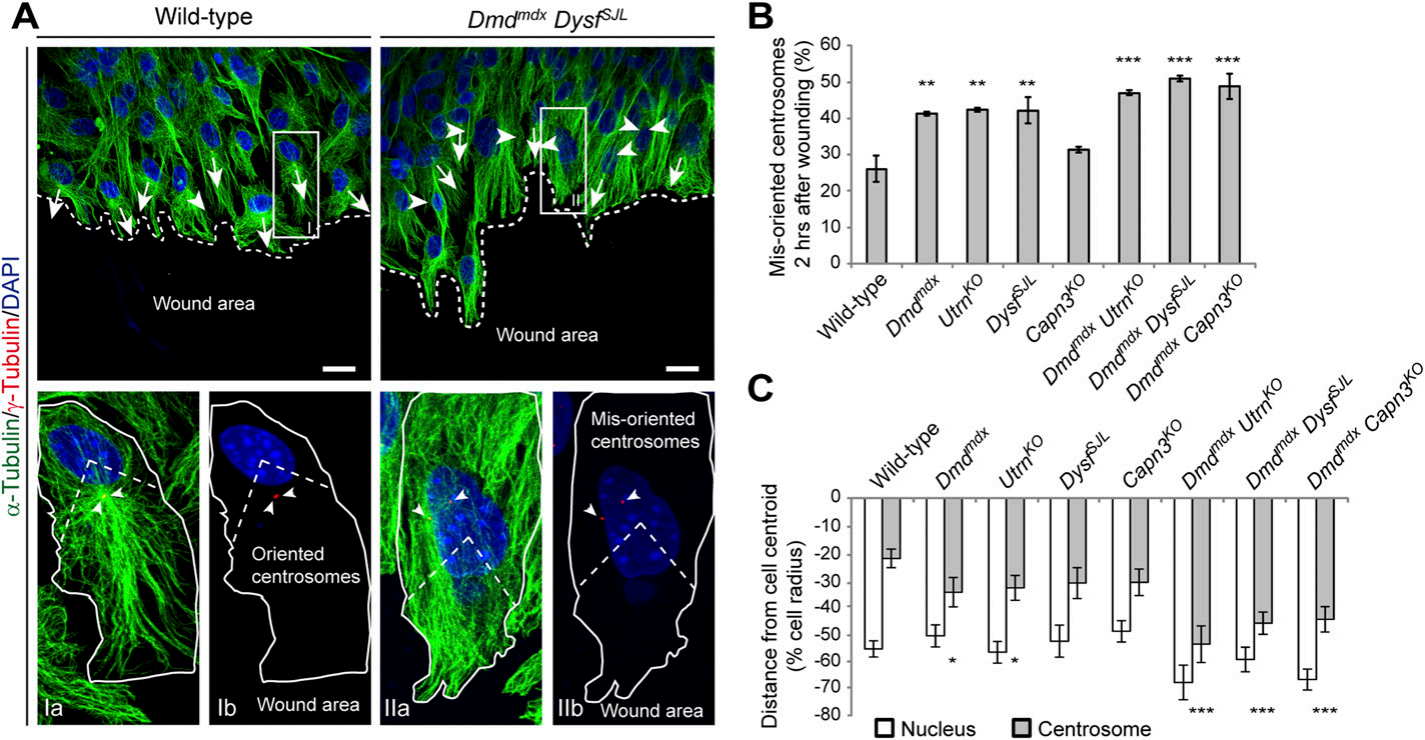
Impaired centrosome reorientation in murine muscular dystrophy myoblasts. **(A)** Representative ICC of microtubules (α-tubulin, green), centrosomes (γ-tubulin, red), and nuclei (DAPI, blue) in WT and *Dmd*^*mdx*^ *Dysf*^*SJL*^ myoblasts in a scratch assay at the edge of a wounded monolayer. Upper panel: white arrows indicate oriented centrosomes; arrowheads indicate misoriented centrosomes; the direction of the arrows/arrowheads denotes the orientation of the respective centrosomes. Boxed areas are shown enlarged in lower panels: Ia and IIa (α-tubulin, γ-tubulin, and DAPI) and Ib and IIb (γ-tubulin and DAPI). White lines outline the periphery of the respective cells, arrowheads indicate the centrosomes, and dashed lines indicate the area between the nucleus and cell periphery oriented toward the wound edge. Centrosomes located within this area were considered as oriented and centrosomes in other parts of the cells as misoriented. Scale bars, 20 μm. **(B)** Statistical analysis of the percentage of oriented centrosomes 2 h after wounding. Mean ± SEM (WT [n = 177], *Dmd*^*mdx*^ [n = 157], *Utrn*^*KO*^ [n = 156], *Dysf*^*SJL*^ [n = 173], *Capn3*^*KO*^ [n = 128], *Dmd*^*mdx*^ *Utrn*^*KO*^ [n = 189], *Dmd*^*mdx*^ *Dysf*^*SJL*^ [n = 181], *Dmd*^*mdx*^ *Capn3*^*KO*^ [n = 178 myoblasts], three newborn mice each). **P < 0.01, ***P < 0.001 (Fisher’s exact test). **(C)** Bar graph shows the position of the nucleus (white) and the centrosome (gray) in WT and muscular dystrophy-mutant myoblasts at the wound edge. Mean ± SEM (WT [n = 42], *Dmd*^*mdx*^ [n = 43], *Utrn*^*KO*^ [n = 40], *Dysf*^*SJL*^ [n = 40], *Capn3*^*KO*^ [n = 43], *Dmd*^*mdx*^ *Utrn*^*KO*^ [n = 39], *Dmd*^*mdx*^ *Dysf*^*SJL*^ [n = 40], *Dmd*^*mdx*^ *Capn3*^*KO*^ [n = 42 myoblasts], three newborn mice each). *P < 0.05, ***P < 0.001 (unpaired *t* test).

Orchestrated reorientation of the centrosome in conjunction with proper nucleation of microtubules in the daughter cell are pivotal mechanisms during cell division. Therefore, we functionally assessed these parameters in WT and MD myoblasts. Upon wounding, reorientation of the centrosomes was significantly disturbed in MD myoblasts (Fig 5A). Although ∼75% of WT myoblasts displayed oriented centrosomes 2 h post-wounding, the proportion of cells with correctly positioned centrosomes was significantly decreased to ∼60% in *Dmd*^*mdx*^, *Utrn*^*KO*^, and *Dysf*^*SJL*^ myoblasts and, again more pronounced, down to ∼50% in all double-mutant MD myoblasts (Fig 5B). It had been shown that upon wounding, myoblasts position their centrosomes between the nucleus and the leading edge to obtain the required migratory front-rear polarity (Chang et al, 2015). Therefore, we evaluated whether the nuclear positioning in relationship with the centrosome localization was affected in MD myoblasts by measuring the distances from the centroid of the nucleus and from the centrosome relative to centroid of the cell (Chang et al, 2016). Although the positions of the nuclei were unaltered in MD myoblasts compared with WT cells, centrosomes were misaligned to a more rearward position in *Dmd*^*mdx*^, *Utrn*^*KO*^, *Capn3*^*KO*^ single mutants and, even more pronounced, in all double-mutant MD myoblasts (Fig 5C).

**Figure 6.**
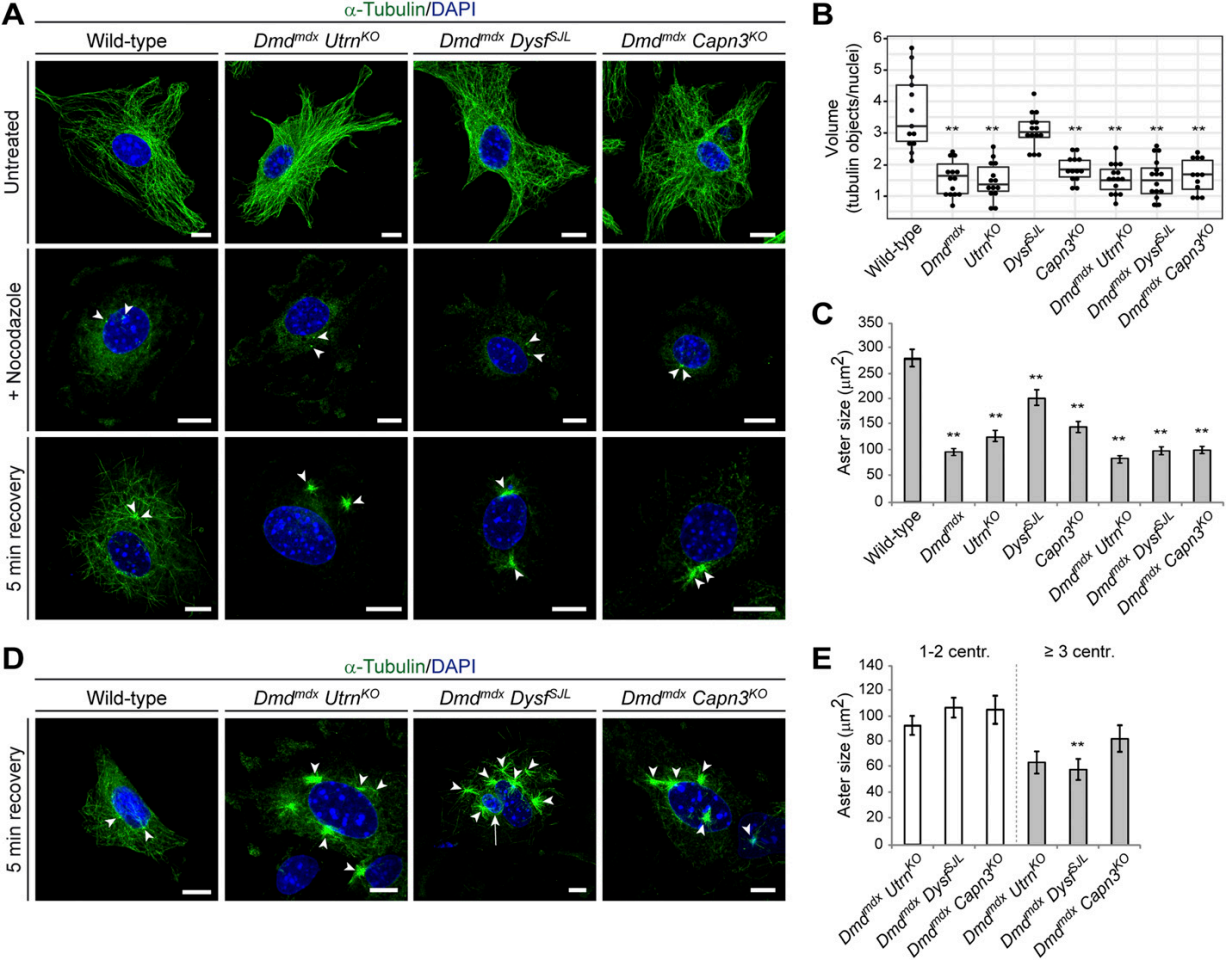
Impaired microtubule regrowth in murine muscular dystrophy myoblasts. **(A)** Representative ICC of microtubules (α-tubulin, green) and nuclei (DAPI, blue) in WT and double-mutant myoblasts, either left untreated, immediately after nocodazole treatment (+nocodazole) or after 5 min of recovery. Arrowheads indicate microtubule organizing centers. Note the severely impaired microtubule regrowth in double-mutant muscular dystrophy myoblasts compared with WT myoblasts. Scale bars, 10 μm. **(B)** Box plots depicting the volumes obtained from α-tubulin signals per volumes of DAPI-positive nuclear area. Each dot represents data from one image stack. **P < 0.01 (one-way ANOVA and post hoc Tukey correction). **(C)** Statistical analysis of the microtubule aster size after 5 min of recovery. Mean ± SEM (WT [n = 89], *Dmd*^*mdx*^ [n = 83], *Utrn*^*KO*^ [n = 89], *Dysf*^*SJL*^ [n = 89], *Capn3*^*KO*^ [n = 89], *Dmd*^*mdx*^ *Utrn*^*KO*^ [n = 89], *Dmd*^*mdx*^ *Dysf*^*SJL*^ [n = 82], *Dmd*^*mdx*^ *Capn3*^*KO*^ [n = 88 centrosomes], three newborn mice each). **P < 0.01 (one-way ANOVA and post hoc Tukey correction). **(D)** Representative ICC images of microtubules (α-tubulin, green), emerging from two centrosomes in a WT control cell or from supernumerary centrosomes in *Dmd*^*mdx*^ *Utrn*^*KO*^, *Dmd*^*mdx*^ *Dysf*^*SJL*^, or *Dmd*^*mdx*^ *Capn3*^*KO*^ myoblasts and nuclei (DAPI, blue), after 5 min of recovery from nocodazole treatment. Arrowheads indicate centrosomes, and arrow denotes a micronucleus. Scale bars, 10 μm. **(E)** Statistical analysis of the microtubule aster size emerging from 1 to 2 centrosomes or from supernumerary centrosomes in *Dmd*^*mdx*^ *Utrn*^*KO*^, *Dmd*^*mdx*^ *Dysf*^*SJL*^, or *Dmd*^*mdx*^ *Capn3*^*KO*^ myoblasts after 5 min of recovery. Mean ± SEM [*Dmd*^*mdx*^ *Utrn*^*KO*^] (n = 65 asters from cells with 1–2 centrosomes, n = 17 asters from cells with supernumerary centrosomes), *Dmd*^*mdx*^ *Dysf*^*SJL*^ (n = 66 asters from cells with 1–2 centrosomes, n = 14 asters from cells with supernumerary centrosomes), *Dmd*^*mdx*^ *Capn3*^*KO*^ (n = 65 asters from cells with 1–2 centrosomes, n = 24 asters from cells with supernumerary centrosomes). **P < 0.01 (unpaired *t* test).

Next, we determined the microtubule-nucleating capacity of centrosomes in murine MD and WT myoblasts by disrupting microtubule assembly by nocodazole (Fig 6A). Microtubule outgrowth after nocodazole removal was significantly impaired in *Dmd*^*mdx*^, *Utrn*^*KO*^, and *Capn3*^*KO*^ single mutants and, even more severe, in all double-mutant MD myoblasts reduced to ∼40–50% of WT levels, indicating perturbed microtubule nucleation arising from centrosomes (Fig 6B and C). Because it has been shown that supernumerary centrosomes directly promote chromosome mis-segregation (Ganem et al, 2009), we tested whether the excess centrosomes, which we have found in MD myoblasts were capable of nucleating microtubules. Indeed, we observed that microtubule outgrowth arises also from supernumerary centrosomes in microtubule-regrowth experiments (Fig 6D). Interestingly, the microtubule asters in MD myoblasts with supernumerary centrosomes appeared smaller than those in cells with 1–2 centrosomes, suggesting that MD-related proteins are indispensable for timely and proper microtubule nucleation (Fig 6E).

## Discussion

There is growing evidence that aberrant expression of dystrophin, utrophin, dysferlin, and calpain-3 does not only give rise to different forms of MDs but is also causatively related to tumorigenesis in humans and mice, which makes MD-related proteins TS candidates (Chamberlain et al, 2007; Korner et al, 2007; Li et al, 2007; Fernandez et al, 2010; Schmidt et al, 2011; Wang et al, 2014; Luce et al, 2017; Gallia et al, 2018; Juratli et al, 2018). Although case reports of cancer in MD patients are rare, growing evidence implicates all four MD genes and/or their protein products in tumorigenesis (Fanzani et al, 2013; Jones et al, 2021), likely by generating a permissive environment for tumor establishment. Especially for *DMD* gene mutations and/or expression changes, numerous reports highlight a clear role in the pathogenesis in a wide range of cancers, including sarcomas, carcinomas, melanomas, lymphomas, and leukemia, as well as brain tumors (Korner et al, 2007; Wang et al, 2014; Jones et al, 2021). To date, 13 DMD cases with cancer have been published, with a high incidence of rhabdomyosarcomas (six patients) being reported (Jones et al, 2021; Vita et al, 2021). In addition, reports of several patients with soft-tissue sarcomas indicate a role of the *DMD* gene and especially of intragenic deletions, in the development of this type of cancer (Wang et al, 2014; Jones et al, 2021). Moreover, reduced *DYSF* mRNA expression levels were found in pancreatic tumors, compared with healthy adjacent tissues, and intronic SNPs in the *DYSF* gene were associated with a higher risk of death (Tang et al, 2017). Interestingly, patients with renal-cell carcinoma and high *DYSF* gene expression levels presented with a better survival rate compared with renal-cell carcinoma patients with low *DYSF* expression (Ha et al, 2019). These results were, however, in contrast to another study, in which *DYSF* mRNA and protein expression levels were oppositely involved in tumor progression (Cox et al, 2020). *CAPN3* expression was found to be reduced in human melanoma cell lines and biopsies, and its overexpression induced p53 stabilization and other effects, ultimately resulting in decreased cell proliferation (Moretti et al, 2009, 2015). Finally, Zhou et al recently showed that reduced *UTRN* expression levels in melanoma patients were associated with advanced clinical characteristics, including decreased survival and poorer prognosis (Zhou et al, 2021). In addition, up-regulated utrophin expression inhibited melanoma cell proliferation (Zhou et al, 2021). These clinical reports are in line with the observation that in MD mice, sarcomas share nonrandom genomic alterations including frequent loss of TS (such as *Cdkn2a* or *Nf1*), amplifications of oncogenes (*Met*, *Jun*), recurrent duplications of whole chromosomes 8 and 15, and DNA damage (Schmidt et al, 2011). However, the functional basis underlying the proposed TS properties of MD-related proteins remains elusive. Our finding that all four MD-related proteins investigated in this study show a centrosomal localization supports are in favor of a common pathomechanistic concept that essentially involves a common function at the centrosome. Therefore, one might hypothesize that also other proteins which are related to both MD and cancer could represent centrosomal proteins in addition to their known (muscle-related) function. To this end, we tested also α-sarcoglycan, as defective expression of which causes MD in humans (LGMDR3) and in mice and, in addition, confers cancer susceptibility in respective *Sgca*^*KO*^ mice (Roberds et al, 1994; Fernandez et al, 2010). In line with our initial hypothesis that proteins related to both MD and cancer are components of the centrosome, we found that α-sarcoglycan also locates to the centrosome by ICC and by probing in centrosome-enriched fractions by WB (data not shown).

Although we found all four MD proteins investigated herein associated with centrosomes in undifferentiated myoblasts, they are differentially expressed and localized during myogenic differentiation. Dystrophin and dysferlin are up-regulated and recruited to the myotube membrane (Belkin & Burridge, 1995) and to the T-tubule system (Klinge et al, 2007) in differentiating C2C12 cells, respectively, whereas utrophin is down-regulated during muscle fiber formation (Galvagni et al, 2002). When embryonic chick myogenic cells were differentiated into multinucleated myotubes, calpain-3 was found in a perinuclear organization, in adhesion structures, and in long-stress fiber-like structures (de Andrade Rosa et al, 2020). In their publication, de Andrade Rosa et al (2020) hypothesized that the perinuclear concentration of calpain-3 in chick muscle cells likely represents the presence of the scaffolding protein calpain-3 in centrosomes and suggest a possible role of calpain-3 in signaling pathways during myogenesis. Whether such an interaction in differentiated myotubes is also feasible for the other MD proteins remains to be investigated.

Our finding that the centrosomal localization of MD-related proteins is not restricted to cells of the myogenic lineage is in line with the finding that defective expression of MD-related proteins is not only restricted to myogenic tumors but has also been shown to be related to different tissue types of non-myogenic cancers (Korner et al, 2007; Li et al, 2007; Schmidt et al, 2011; Fanzani et al, 2013; Wang et al, 2014; Luce et al, 2017; Gallia et al, 2018; Juratli et al, 2018; Jones et al, 2021). Remarkably, our finding that the MD proteins we investigated in this study are expressed in different suborganellar compartments of the centrosome support the notion that they are engaged in different functional pathways with respect to centrosome-related functions, which has to be tested in further experiments. We show here that the absence of MD-related proteins leads to centrosome amplification in murine and human myoblasts, which is consistent with the findings of Dumont et al (2015), who found centrosome amplification in dystrophin-deficient muscle stem cells (satellite cells, SCs; Dumont et al, 2015). Moreover, they showed that in SCs, dystrophin contributes to SC polarity and asymmetric division via association with Mark-2, an important regulator of cell polarity (Dumont et al, 2015). In the absence of dystrophin, expression of Mark-2 is down-regulated, resulting in a strikingly reduced number of asymmetric divisions, leading to loss of polarity, impaired mitotic spindle orientation, prolonged cell divisions, and abnormal division parameters including centrosome amplification (Dumont et al, 2015). In another study, it has been shown that the activation of epidermal growth factor receptor through Aurora kinase A (Aurka) regulates orientation of centrosomes during asymmetric SC division (Wang et al, 2019). Ultimately, in vivo EGF treatment in *Dmd*^mdx^ mice rescued the reduction of asymmetric divisions in dystrophin-deficient SCs and resulted in increased numbers of progenitors and enhanced regeneration, thus restoring muscle strength (Wang et al, 2019). Whether this signaling pathway also plays a role in SCs from other MD mouse models, such as *Utrn*^KO^, *Dysf*^SJL^, or *Capn3*^KO^, remains to be investigated.

Multiple centrosome abnormalities represent a hallmark of virtually all cancer types and have been linked to chromosomal instability and tumorigenesis (Cosenza & Kramer, 2016). In vitro, supernumerary centrosomes have been shown to promote chromosome mis-segregation during cell division (Ganem et al, 2009) and favor invasive phenotypes in a 3D-culture model (Godinho et al, 2014). Moreover, the presence of supernumerary centrosomes per se seems to be sufficient to drive aneuploidy and the development of various kinds of spontaneous tumors, including sarcomas (Levine et al, 2017). Therefore, it is tempting to speculate that the centrosome amplification that we have observed in human and murine MD myoblasts might be causatively implicated in genomic instability and DNA damage, which we have previously found in dystrophic muscle of MD mice and in muscles and myoblasts of human MD patients (Schmidt et al, 2011). Moreover, the acquisition of supernumerary centrosomes and the generation of multipolar spindles during mitosis likely facilitate unequal chromatin segregation during cell division (Nigg, 2002; Raff & Basto, 2017), which might ultimately lead to nuclear abnormalities. In line with this concept, we observed that human and murine myoblasts lacking the expression of the proteins studied in this study are frequently multinucleated and display numerous nuclear abnormalities such as micronuclei and nuclear blebs. Moreover, centrosome amplification and nuclear abnormalities were even more increased in myoblasts from double-mutant *Dmd*^*mdx*^ *Utrn*^*KO*^, *Dmd*^*mdx*^ *Capn3*^*KO*^, or *Dmd*^*mdx*^ *Dysf*^*SJL*^ mice, highly suggestive of a negative additive effect of MD protein deficiencies with respect to these abnormalities. In general, mispositioning of myonuclei is a common feature of many MDs, and there is increasing evidence that mispositioned myonuclei are not merely a symptom but also drivers of dystrophic changes in MDs (Folker & Baylies, 2013). Moreover, nuclear positioning is closely associated with centrosome orientation in myoblasts and has important pathomechanistic consequences during directional cell migration as it is pivotal for establishing cell polarity (Chang et al, 2015).

Our findings suggested that loss of MD-related proteins can impede adequate reorientation and rearward positioning of centrosomes during migration, whereas proper nuclear positioning was unaffected. Also in this context, we provide evidence that MD-related proteins act cooperatively by demonstrating that double-mutant MD myoblasts display a significantly lower fraction of cells with properly orientated centrosomes as compared with single-mutant cells. We also show that MD-related proteins are involved in conferring timely and proper microtubule nucleation because MD myoblasts display aberrant and impaired regrowth of microtubules in a respective assay. Also with respect to this phenotype, cooperativity of the MD-related proteins is suggested because the outgrowth was dramatically hampered in double-mutant myoblasts as compared with single mutants. Our findings that MD-related proteins are implicated in two dynamic centrosome-related processes, that is, migration-dependent centrosome reorientation and, in addition, conferring orchestrated microtubule outgrowth, indicates a functional role of these proteins in centrosome biology in more general. However, it should be noted that our data derived from nocodazole treatment experiments cannot discriminate between defective nucleation and outgrowth dynamics, representing an important limitation of this study, which needs to be addressed in future work. At least dystrophin behaves like a microtubule-associated protein by interacting with microtubules in skeletal-muscle cells (Prins et al, 2009). Dysferlin also interacts with α-tubulin and microtubules (Azakir et al, 2010), preventing microtubule depolymerization by controlling the levels of α-tubulin acetylation in myoblasts (Di Fulvio et al, 2011). Contrary to dystrophin, which binds to microtubules with high affinity and pauses microtubule polymerization, utrophin has been shown to be inactive in microtubule binding assays and rescue experiments (Belanto et al, 2014). Moreover, even though no interaction between calpain-3 and tubulin has been reported so far, calpain-3 was reported to act as a modulator of the dysferlin protein complex (Huang et al, 2008) and could probably thereby influence the cytoskeleton. Although microtubules and their associated proteins and motors mediate most of the nuclear movements studied to date (Luxton et al, 2011; Luxton & Gundersen, 2011), it has been shown that nuclear movement and positioning as well as centrosome orientation in migrating myoblasts also require the actin cytoskeleton and its associated factors (Chang et al, 2015). Indeed, γ-actin is also implicated in regulating centrosome function and mitotic progression in cancer cells (Po’uha & Kavallaris, 2015) and therefore represents another candidate for interaction with MD proteins. In most cellular contexts, the actin network and the microtubules are intimately connected and often co-regulated, making it difficult to discern the specific effects of either network (Rodriguez et al, 2003). As all four MD-related proteins studied herein interact with the cytoskeleton in various ways, it might be anticipated that additional pathways contribute to the observed pathologic phenotypes.

Even though we present a hitherto unreported centrosomal localization of several MD proteins together with a pathological phenotype in MD protein-lacking cells, the causative pathomechanism interlinking cancerogenesis and muscle damage has not yet been identified. First, insights gained by centrosome reorientation and microtubule nucleation experiments open a perspective for a possible function, but more in-depth studies will be needed to answer these questions. Strikingly, all centrosome-related pathologies which we show here, namely, the occurrence of supernumerary centrosomes, nuclear abnormalities, impaired orientation, and microtubule re-polymerization, were markedly aggravated in double-mutant MD myoblasts. This additive effect on the cellular level thus could explain why combined defects in MD genes provoked dramatically increased frequency and the earlier onset of sarcoma formation in *Dmd*^*mdx,*^ *Dysf*^*SJL*^ (Schmidt et al, 2011), *Dmd*^*mdx-5Cv*^ *Dysf*^*prmd*^ (Hosur et al, 2012), and *Dmd*^*mdx*^ *Capn3*^*KO*^ (Schmidt et al, 2011) double-mutant MD mice.

Up to now, lack of utrophin was not linked to any severe phenotype in respective knockout mice. In humans, however, defective expression of utrophin because of *UTRN* mutations has been detected in various types of human tumors, such as breast cancers, neuroblastomas, and malignant melanomas (Li et al, 2007). Here, we describe for the first time that the absence of utrophin gives rise to centrosome-related phenotypes on the cellular level, some of which were even more pronounced as compared with MD myoblasts deficient for dystrophin, dysferlin, or calpain-3. Noteworthy, the combined loss of utrophin and dystrophin gives rise to a markedly aggravated centrosome-related phenotype, suggesting an additive interaction of both proteins on the cellular level (Schmidt et al, 2011). Therefore, it is tempting to speculate that this might reflect the underlying pathomechanism responsible for the aggravation of the MD-related phenotype in double-mutant (*Dmd*^*mdx*^ *Utrn*^*KO*^) MD mice (Deconinck et al, 1997b).

In conclusion, we identified for the first time a centrosomal localization of four different MD-related proteins, dystrophin, utrophin, dysferlin, and calpain-3, in myoblasts and non-muscle cells and show that their absence leads to extra centrosomes, impaired centrosome function, and abnormal nuclear morphology. Therefore, we introduce a novel pathomechanistic concept that will foreseeably help to better understand the emerging link between MDs and cancer.

## Materials and Methods

### Animals

Mouse stocks were maintained at the Division for Laboratory Animal Science and Genetics, Medical University of Vienna, according to Austrian Federal Government laws and regulations. The following mouse lines were used in this study: *mdx* (*Dmd*^*mdx*^) mice (Bulfield et al, 1984) and dysferlin-deficient (*Dysf*^*SJL*^, because of the *SJL* mutation) mice bred on a C57BL/10J background (Bittner et al, 1999) were originally obtained from the Jackson Laboratory; *Capn3*^*tm1Isdr*^ (*Capn3*^*KO*^) mice (Richard et al, 2000) were obtained from Isabelle Richard; *Utrn*^*tm1Ked*^ (*Utrn*^*KO*^) mice (Deconinck et al, 1997a) were obtained from Kay E Davies. All mice were inbred on a C57BL/10J background (WT; Jackson Laboratory). To obtain double-mutant mice (*Dmd*^*mdx*^ *Utrn*^*KO*^, *Dmd*^*mdx*^ *Dysf*^*SJL*^, or *Dmd*^*mdx*^ *Capn3*^*KO-*^), *Dmd*^*mdx*^ mice were crossed with *Utrn*^*KO*^, *Dysf*^*SJL*^, or *Capn3*^*KO*^ mice.

### Cell culture

C2C12 cells (European Collection of Authenticated Cell Cultures, ECACC 91031101) were grown in proliferation medium (DMEM [Gibco]) supplemented with 20% FCS (Sigma-Aldrich), 2 mM L-glutamine (Gibco), 50 U/ml penicillin, and 50 μg/ml streptomycin (P/S; Gibco) at 37°C in a humidified atmosphere of 5% CO_2_. Mouse fibroblasts (*p53*^*−/−*^) (Andrä et al, 2003; Winter et al, 2008), human WI-38 fibroblasts (ECAAC 90020107), HeLa cells (ECACC 93021013), and Hep G2 cells (ECACC 85011430) were grown in proliferation medium (DMEM supplemented with 10% FCS, 2 mM L-glutamine, and P/S) at 37°C in a humidified atmosphere of 5% CO_2_.

Primary mouse myoblasts were isolated from neonatal mice (1–2 d) as described previously (Winter et al, 2014). De-skinned front and hind limbs were enzymatically dissociated in 3 ml enzyme solution (0.2% collagenase I in serum-free DMEM medium containing 100 nM nonessential amino acids [NEAA, Gibco], 2 mM L-glutamine, P/S) for 1.5–2 h at 37°C with gentle agitation. The digested tissue was poured into 5 ml pre-warmed medium (serum-free DMEM supplemented with NEAA, P/S, and L-glutamine), and single muscle fibers were released by gentle trituration with a glass pipette. The slurry was collected by centrifugation and washed twice in PBS (15*g* for 3 min). The pellet was resuspended in 2.5 ml DMEM containing 20% FCS, 10% horse serum (Gibco), 1% chicken embryo extract (Seralab), and P/S and plated on Geltrex (Geltrex LDEV-Free Reduced Growth Factor Basement Membrane Matrix, Thermo Fisher Scientific; diluted 1:100 in DMEM)-coated Ø 10-cm dishes for 48 h at 37°C in a humidified atmosphere of 5% CO_2_. Myoblasts were split, pre-plated on uncoated culture dishes for up to 2 h (to remove contaminating fibroblasts), and cultivated in Ham’s F10 medium (Gibco) supplemented with 20% FCS, 2.5 ng/ml basic fibroblast growth factor (Promega), and P/S on collagen-coated (0.01% collagen [PureCol; CellSystems] in PBS) culture dishes. For ICC, primary mouse myoblasts were cultivated on Geltrex-coated eight-well μ slides (Ibidi) for 24 h before fixation.

Primary human myoblasts were obtained from the Muscle Tissue Culture Collection, Friedrich-Baur-Institute, Department of Neurology, Ludwig-Maximilian-University Munich. DMD: “Essen 88/07” (age at biopsy: 14 yr (a), del45_50); “72/05” (7 a, dup_ex8-29); “Essen 8/02” (4 a, del_ex51-55); “166/00” (6 a, 2-bp deletion in exon 6); LGMDR2: “90/01” (36 a, female, c.[638C>T];[5249delG]); “176/01” (32 a, male, c.[2367C>A];[5979dupA]); “362/03” (male, 33 a, c.[exon 5 p.Pro134Leu];[5022delT]); controls: “363/07” (21a, male); “179/07” (21 a, female). Cells were cultivated in skeletal-muscle cell growth medium (PromoCell) containing P/S at 37°C in a humidified atmosphere of 5% CO_2_.

### Antibodies

The following primary antibodies were used for ICC and/or WB: rabbit antiserum (AS) to dystrophin #2166 (ICC 1:200; directed against the last 17 amino acids of murine dystrophin; Blake et al, 1999), ab15277 (ICC 1:500; Abcam), sheep AS to dystrophin (ICC 1:5,000, 60 kD; Hoffman et al, 1987), mouse mAbs to dystrophin DYS1 (ICC 1:100, WB 1:2,000; NCL-DYS1; Novocastra), DYS2 (ICC 1:100, WB 1:400; NCL-DYS2; Novocastra), DYS3 (ICC 1:100; NCL-DYS3; Novocastra), and MANDRA1 (ICC 1:100, WB 1:400; D8043; Sigma-Aldrich), mouse mAbs to utrophin (WB: 1:1,000; NCL-DRP2; Novocastra), goat AS to utrophin (ICC 1:100; N19, sc-7460; Santa Cruz), mouse mAbs to dysferlin (ICC 1:50, WB 1:900; NCL-Hamlet; Novocastra), rabbit AS to calpain-3 (ICC 1:100; directed against the IS2 region; Baghdiguian et al, 1999; gift from I Richard), mouse mAbs to calpain-3 (WB 1:100; NCL-CALP-12A2; Novocastra), rabbit AS to γ-tubulin (ICC 1:200, WB 1:1.500; ab11317; Abcam), mouse mAbs to γ-tubulin (ICC 1:200, WB 1:1,000; clone GTU-88; Sigma-Aldrich), mouse mAbs to α-tubulin (ICC 1:1,000, WB 1:1,000; T5168; Sigma-Aldrich), rabbit AS to GAPDH (WB 1:10,000; G9545; Sigma-Aldrich), and mouse mAbs to centrin-1 (ICC 1:100; 04-1624; Merck Millipore). For ICC, primary antibodies were used in combination with donkey anti-mouse IgG Alexa 488, donkey anti-mouse IgG Alexa 594, donkey anti-rabbit IgG Alexa 488, donkey anti-rabbit IgG Alexa 594, and donkey anti-sheep/goat Alexa 594 (all from Invitrogen). For immunoblot analyses, HRP-conjugated secondary antibodies were used (Dako) in combination with ECL Select WB detection reagent (Amersham).

### Immunocytochemistry (ICC)

Cells were fixed with ice-cold methanol for 2 min at −20°C, washed with PBS, and blocked with 5% BSA (Pan Biotech GmbH) in PBS for 1 h at room temperature. Next, cells were incubated with primary antibodies diluted in PBS over night at 4°C in a humidified chamber. For dystrophin-specific immunostaining, either monoclonal DYS1, DYS2 (both from Leica Biosystems), and MANDRA1 antibodies (Sigma-Aldrich) were combined, or polyclonal antisera were used to enhance the number of dystrophin epitopes (see also Fig S1A and B). After washing with PBS, cells were incubated with secondary antibodies for 2 h at room temperature and again washed with PBS. Nuclei were stained with 4,6-diamidin-2′-phenylindol-dihydrochlorid (DAPI; Sigma-Aldrich). C2C12 cells and primary human myoblasts, grown on coverslips, were mounted in Mowiol 4–88 supplemented with 2.5 g/100 ml 1,4-diazabicyclo[2.2.2]octane (both from Sigma-Aldrich); primary mouse myoblasts, which were grown on eight-well μ slides, were mounted in Ibidi mounting medium (Ibidi). Microscopy was performed using an Olympus FLUOVIEW FV3000 confocal microscope equipped with PlanApo N 60× 1.4 NA and UPLAN FLN 40× 1.3 NA objective lenses (Olympus). Images were recorded using the Olympus FluoView software and processed and analyzed using ImageJ software (NIH). Co-localization analysis (Pearson’s correlation coefficient, Mander’s co-localization coefficients) of fluorescence signals was performed from confocal maximum intensity projections using “Coloc2” in ImageJ.

### Preparation of cell and tissue lysates, SDS–PAGE, and WB analysis

Cells grown on a 15-cm dish were directly scraped off in 200 μl lysis buffer (pH 7.5) containing 150 mM NaCl, 2M urea, 20 mM Tris, 2 mM EDTA, 2 mM EGTA, 3.5% SDS, and 1.5% β-mercaptoethanol, mixed with 6× SDS sample buffer (500 mM Tris–HCl [pH 6.8], 600 mM DDT, 10% SDS, 0.1% bromophenol-blue, and 30% glycerol), DNA sheared by pressing the samples through a 27-gauge needle, and samples were incubated for 5 min at 95°C (Winter et al, 2014). Serial 5-μm cryosections of frozen skeletal-muscle tissue (quadriceps femoris) were homogenized in lysis buffer, mixed with 6× SDS sample buffer, and incubated for 5 min at 95°C. SDS–PAGE was performed as described (Laemmli, 1970). Proteins were transferred to nitrocellulose membranes (Protran 0.45 NC; Amersham) using a Mini-PROTEAN Tetra Cell blot apparatus (Bio-Rad).

### RNA isolation

RNA was isolated from serial 10-μm cryosections of frozen skeletal muscle or from a Ø 10-cm dish of confluent cells by lysis in 1 ml TRIZOL reagent (800 mM guanidine thiocyanate, 400 mM ammonium thiocyanate, 100 mM sodium acetate, 5% glycerol, and 38% phenol in RNase-free water), followed by chloroform extraction and precipitation with isopropanol. RNA samples were measured by spectrophotometry (NanoDrop), and integrity was confirmed by electrophoretic separation on agarose gels.

### Nested reverse-transcriptase (RT) PCR

To analyze whether C2C12 myoblasts, mouse *p53*^*−/−*^ fibroblasts, human WI-38 fibroblasts, or HeLa cells express dystrophin, isolated RNA was subjected to RT–PCR. RNA (200 ng) was reverse-transcribed using specific priming with an antisense oligonucleotide targeting exon 12 of dystrophin (primer sequence [5′->3′] for mouse *Dmd* was GGC TCT TCC TCC ATT TTC TTA GTT and for human *DMD* GTT GTA CTT GGC GTT TTA GGT CTT) in a 20 μl reaction containing 1 μM primer, 4 μl 5× first strand reaction buffer (Thermo Fisher Scientific), 0.5 mM dNTPs, 10 mM DTT, and 50 U Superscript II (Thermo Fisher Scientific). cDNA synthesis was performed for 60 min at 42°C and terminated by incubation at 70°C for 10 min. Amplification was obtained by two rounds of PCR (using the GoTaq DNA Polymerase protocol; Promega) using the following primer pairs (0.5 μM each): first round PCR included primers complementary to the sequences of 5′UTR and exon 11 (mouse *Dmd* gene: m5′UTR, GTT TAT TGG CTT CTC ATC GTA CCT; mEx11rev, CTT CTG ATA ATT TCC CTT TTC CAA; human *DMD* gene: h5′UTR, TGC TGA AGT TTG TTG GTT TCT CAT; hEx11rev, ATT TTC CTG TTC CAA TCA GCT TAC). After cycling (3 min 95°C, 6× [40 s 95°C, 40 s 60°C, 90 s 72°C], 32× [30 s 95°C, 30 s 60°C, 90 s 72°C], 5 min 72°C), 1 μl of a 1:100 dilution of first-round PCR reaction products were used as the template for nested PCR. Second-round primers were designed complementary to exons 1 and 10 of the dystrophin gene (mouse *Dmd* gene: mEx1for, GGT GGG AAG AAG TAG AGG ACT GT; mEx10rev, CAT CAT TTG AAA TCT CTC CTT GTG; human *DMD* gene: hEx1for, GCT TTG GTG GGA AGA AGT AGA GGA CTG T; hEx10rev, GTC CAG GTT TAC TTC ACT CTC CAT). PCR products were separated in Midori Green-stained agarose gels, excised, and purified with the illustra GFX PCR DNA and Gel Band Purification Kit (GE Healthcare). Capillary DNA sequencing was performed at Eurofins Genomics, Austria.

### Preparation of centrosomes

Centrosomes were isolated from confluent C2C12 cells essentially as reported (Gogendeau et al, 2015). Cells were scraped off in growth medium and treated with 10 μg/ml nocodazole and 2 μM cytochalasin D (both from Sigma-Aldrich) for 1 h at 37°C to depolymerize microtubules and actin filaments. After centrifugation at 280*g* for 8 min, cells were washed sequentially in 1× TBS (Tris-buffered saline) and 0.1× TBS/8% sucrose and then carefully lysed in 1 mM Hepes (pH 7.2), 0.5% Igepal, 0.5 mM MgCl_2_, 0.1% β-mercaptoethanol, and Complete Inhibitor Cocktail tablets (Roche) for 5 min on ice. After centrifugation at 2,500*g* for 10 min, the lysis supernatant was filtered through a 40-μm nylon mesh and incubated with 20 U/ml DNase I (AppliChem) and 10 mM Hepes (pH 7.2) for 30 min at 4°C. The lysate was underlaid with 60% sucrose solution (w/w) in gradient buffer (10 mM Pipes [pH 7.2], 0.1% Triton X-100, 0.1% β-mercaptoethanol) and centrifuged at 10,400*g* for 30 min. The interface containing centrosomes and sucrose cushion was collected and further purified on a discontinuous (70, 50, and 40%) sucrose gradient by centrifugation at 120,000*g* for 1.5 h. For detection of dystrophin by WB analysis, fractions were concentrated three- to fourfold by using Amicon Ultra Centrifugal Filters (Merck Millipore).

### Centrosome reorientation assay

Centrosome reorientation after wounding was assessed as described (Palazzo et al, 2001; Chang et al, 2015). Primary mouse myoblasts were serum-starved in DMEM containing L-glutamine, P/S, and 0.1% FCS for 24 h, wounded with a 200-μl pipette tip, and reorientation of centrosomes was stimulated by incubation in fresh proliferation medium for 2 h at 37°C. Afterward, cells were fixed with ice-cold methanol and stained with α- and γ-tubulin antibodies for ICC. Centrosome orientation and nuclear and centrosomal positions were measured using ImageJ as described (Chang et al, 2016).

### Microtubule regrowth assay

The microtubule regrowth assay was performed as described (Delgehyr et al, 2005; Fumoto et al, 2009). Primary mouse myoblasts were treated with 5 μM nocodazole in proliferation medium for 1 h at 37°C. Afterward, cells were washed, incubated for 5 min with fresh proliferation medium to allow microtubule regrowth, fixed with ice-cold methanol, and stained with α-tubulin antibodies for ICC. Analysis of microtubule regrowth was performed using ImageJ v1.52n by importing image stack files (*.oir) using the Bio-Formats Macro Extensions. After thresholding each channel (using the “Huang dark stack” method), the 3D Objects Counter function was used to automatically detect objects. Total size of green fluorescence objects were normalized to total size of DAPI objects for each image stack. Aster size was measured using ImageJ as well.

### Quantification and statistical analysis

Experiments were performed at least in biological triplicates, unless otherwise stated. Data are presented as the mean ± SEM or median with minimum and maximum (whiskers). Statistical analysis was performed using Excel or GraphPad statistical software. Comparisons of categorical data between two groups were made using a two-tailed Fisher’s exact test. Comparisons among values of multiple groups were performed using one-way ANOVA. The significance between the individual groups was subsequently determined using the Tukey post hoc test (α = 0.05).

## Supplementary Material

Reviewer comments

## References

[bib1] Andrä K, Kornacker I, Jörgl A, Zörer M, Spazierer D, Fuchs P, Fischer I, Wiche G (2003) Plectin-isoform-specific rescue of hemidesmosomal defects in plectin (-/-) keratinocytes. J Invest Dermatol 120: 189–197. 10.1046/j.1523-1747.2003.12027.x 12542521

[bib2] Azakir BA, Di Fulvio S, Therrien C, Sinnreich M (2010) Dysferlin interacts with tubulin and microtubules in mouse skeletal muscle. PLoS One 5: e10122. 10.1371/journal.pone.0010122 20405035 PMC2853571

[bib3] Baghdiguian S, Martin M, Richard I, Pons F, Astier C, Bourg N, Hay RT, Chemaly R, Halaby G, Loiselet J, (1999) Calpain 3 deficiency is associated with myonuclear apoptosis and profound perturbation of the IκBα/NF-κB pathway in limb-girdle muscular dystrophy type 2A. Nat Med 5: 503–511. 10.1038/8385 10229226

[bib4] Belanto JJ, Mader TL, Eckhoff MD, Strandjord DM, Banks GB, Gardner MK, Lowe DA, Ervasti JM (2014) Microtubule binding distinguishes dystrophin from utrophin. Proc Natl Acad Sci USA 111: 5723–5728. 10.1073/pnas.1323842111 24706788 PMC3992671

[bib5] Belkin AM, Burridge K (1995) Association of aciculin with dystrophin and utrophin. J Biol Chem 270: 6328–6337. 10.1074/jbc.270.11.6328 7890770

[bib6] Bittner RE, Anderson LV, Burkhardt E, Bashir R, Vafiadaki E, Ivanova S, Raffelsberger T, Maerk I, Höger H, Jung M, (1999) Dysferlin deletion in SJL mice (SJL-Dysf) defines a natural model for limb girdle muscular dystrophy 2B. Nat Genet 23: 141–142. 10.1038/13770 10508505

[bib7] Blake DJ, Hawkes R, Benson MA, Beesley PW (1999) Different dystrophin-like complexes are expressed in neurons and glia. J Cell Biol 147: 645–658. 10.1083/jcb.147.3.645 10545507 PMC2151186

[bib8] Bulfield G, Siller WG, Wight PA, Moore KJ (1984) X chromosome-linked muscular dystrophy (mdx) in the mouse. Proc Natl Acad Sci USA 81: 1189–1192. 10.1073/pnas.81.4.1189 6583703 PMC344791

[bib9] Chamberlain JS, Metzger J, Reyes M, Townsend D, Faulkner JA (2007) Dystrophin-deficient mdx mice display a reduced life span and are susceptible to spontaneous rhabdomyosarcoma. FASEB J 21: 2195–2204. 10.1096/fj.06-7353com 17360850

[bib10] Chang W, Antoku S, Gundersen GG (2016) Wound-healing assays to study mechanisms of nuclear movement in fibroblasts and myoblasts. Methods Mol Biol 1411: 255–267. 10.1007/978-1-4939-3530-7_17 27147048

[bib11] Chang W, Antoku S, Ostlund C, Worman HJ, Gundersen GG (2015) Linker of nucleoskeleton and cytoskeleton (LINC) complex-mediated actin-dependent nuclear positioning orients centrosomes in migrating myoblasts. Nucleus 6: 77–88. 10.1080/19491034.2015.1004947 25587885 PMC4615731

[bib12] Cosenza MR, Kramer A (2016) Centrosome amplification, chromosomal instability and cancer: Mechanistic, clinical and therapeutic issues. Chromosome Res 24: 105–126. 10.1007/s10577-015-9505-5 26645976

[bib13] Cox A, Zhao C, Tolkach Y, Nettersheim D, Schmidt D, Kristiansen G, Hauser S, Muller SC, Ritter M, Ellinger J (2020) The contrasting roles of Dysferlin during tumor progression in renal cell carcinoma. Urol Oncol 38: 687.e1-687.e11. 10.1016/j.urolonc.2020.04.021 32430251

[bib14] de Andrade Rosa I, Correa S, Costa ML, Mermelstein C (2020) The scaffolding protein calpain-3 has multiple distributions in embryonic chick muscle cells and it is essential for the formation of muscle fibers. Tissue Cell 67: 101436. 10.1016/j.tice.2020.101436 32932207

[bib15] Deconinck AE, Potter AC, Tinsley JM, Wood SJ, Vater R, Young C, Metzinger L, Vincent A, Slater CR, Davies KE (1997a) Postsynaptic abnormalities at the neuromuscular junctions of utrophin-deficient mice. J Cell Biol 136: 883–894. 10.1083/jcb.136.4.883 9049253 PMC2132499

[bib16] Deconinck AE, Rafael JA, Skinner JA, Brown SC, Potter AC, Metzinger L, Watt DJ, Dickson JG, Tinsley JM, Davies KE (1997b) Utrophin-dystrophin-deficient mice as a model for Duchenne muscular dystrophy. Cell 90: 717–727. 10.1016/s0092-8674(00)80532-2 9288751

[bib17] Delgehyr N, Sillibourne J, Bornens M (2005) Microtubule nucleation and anchoring at the centrosome are independent processes linked by ninein function. J Cell Sci 118: 1565–1575. 10.1242/jcs.02302 15784680

[bib18] Di Fulvio S, Azakir BA, Therrien C, Sinnreich M (2011) Dysferlin interacts with histone deacetylase 6 and increases alpha-tubulin acetylation. PLoS One 6: e28563. 10.1371/journal.pone.0028563 22174839 PMC3234273

[bib19] Doorenweerd N, Mahfouz A, van Putten M, Kaliyaperumal R, t’Hoen PAC, Hendriksen JGM, Aartsma-Rus AM, Verschuuren JJGM, Niks EH, Reinders MJT, (2017) Timing and localization of human dystrophin isoform expression provide insights into the cognitive phenotype of Duchenne muscular dystrophy. Sci Rep 7: 12575. 10.1038/s41598-017-12981-5 28974727 PMC5626779

[bib20] Dumont NA, Wang YX, von Maltzahn J, Pasut A, Bentzinger CF, Brun CE, Rudnicki MA (2015) Dystrophin expression in muscle stem cells regulates their polarity and asymmetric division. Nat Med 21: 1455–1463. 10.1038/nm.3990 26569381 PMC4839960

[bib21] Fanzani A, Monti E, Donato R, Sorci G (2013) Muscular dystrophies share pathogenetic mechanisms with muscle sarcomas. Trends Mol Med 19: 546–554. 10.1016/j.molmed.2013.07.001 23890422

[bib22] Fernandez K, Serinagaoglu Y, Hammond S, Martin LT, Martin PT (2010) Mice lacking dystrophin or alpha sarcoglycan spontaneously develop embryonal rhabdomyosarcoma with cancer-associated p53 mutations and alternatively spliced or mutant Mdm2 transcripts. Am J Pathol 176: 416–434. 10.2353/ajpath.2010.090405 20019182 PMC2797901

[bib23] Folker ES, Baylies MK (2013) Nuclear positioning in muscle development and disease. Front Physiol 4: 363. 10.3389/fphys.2013.00363 24376424 PMC3859928

[bib24] Fumoto K, Kadono M, Izumi N, Kikuchi A (2009) Axin localizes to the centrosome and is involved in microtubule nucleation. EMBO Rep 10: 606–613. 10.1038/embor.2009.45 19390532 PMC2711835

[bib25] Gallia GL, Zhang M, Ning Y, Haffner MC, Batista D, Binder ZA, Bishop JA, Hann CL, Hruban RH, Ishii M, (2018) Genomic analysis identifies frequent deletions of Dystrophin in olfactory neuroblastoma. Nat Commun 9: 5410. 10.1038/s41467-018-07578-z 30575736 PMC6303314

[bib26] Galvagni F, Cantini M, Oliviero S (2002) The utrophin gene is transcriptionally up-regulated in regenerating muscle. J Biol Chem 277: 19106–19113. 10.1074/jbc.m109642200 11875058

[bib27] Ganem NJ, Godinho SA, Pellman D (2009) A mechanism linking extra centrosomes to chromosomal instability. Nature 460: 278–282. 10.1038/nature08136 19506557 PMC2743290

[bib28] Godinho SA, Picone R, Burute M, Dagher R, Su Y, Leung CT, Polyak K, Brugge JS, Thery M, Pellman D (2014) Oncogene-like induction of cellular invasion from centrosome amplification. Nature 510: 167–171. 10.1038/nature13277 24739973 PMC4061398

[bib29] Gogendeau D, Guichard P, Tassin AM (2015) Purification of centrosomes from mammalian cell lines. Methods Cell Biol 129: 171–189. 10.1016/bs.mcb.2015.03.004 26175439

[bib30] Grady RM, Teng H, Nichol MC, Cunningham JC, Wilkinson RS, Sanes JR (1997) Skeletal and cardiac myopathies in mice lacking utrophin and dystrophin: A model for duchenne muscular dystrophy. Cell 90: 729–738. 10.1016/s0092-8674(00)80533-4 9288752

[bib31] Ha M, Jeong H, Roh JS, Lee B, Han ME, Oh SO, Sohn DH, Kim YH (2019) DYSF expression in clear cell renal cell carcinoma: A retrospective study of 2 independent cohorts. Urol Oncol 37: 735–741. 10.1016/j.urolonc.2019.07.007 31377166

[bib32] Helliwell TR, Man NT, Morris GE, Davies KE (1992) The dystrophin-related protein, utrophin, is expressed on the sarcolemma of regenerating human skeletal muscle fibres in dystrophies and inflammatory myopathies. Neuromuscul Disord 2: 177–184. 10.1016/0960-8966(92)90004-p 1483043

[bib33] Hoffman EP, Brown RH Jr., Kunkel LM (1987) Dystrophin: The protein product of the duchenne muscular dystrophy locus. Cell 51: 919–928. 10.1016/0092-8674(87)90579-4 3319190

[bib34] Hosur V, Kavirayani A, Riefler J, Carney LM, Lyons B, Gott B, Cox GA, Shultz LD (2012) Dystrophin and dysferlin double mutant mice: A novel model for rhabdomyosarcoma. Cancer Genet 205: 232–241. 10.1016/j.cancergen.2012.03.005 22682622 PMC3372852

[bib35] Huang Y, de Morree A, van Remoortere A, Bushby K, Frants RR, den Dunnen JT, van der Maarel SM (2008) Calpain 3 is a modulator of the dysferlin protein complex in skeletal muscle. Hum Mol Genet 17: 1855–1866. 10.1093/hmg/ddn081 18334579 PMC2900895

[bib36] Jones L, Naidoo M, Machado LR, Anthony K (2021) The Duchenne muscular dystrophy gene and cancer. Cell Oncol 44: 19–32. 10.1007/s13402-020-00572-y PMC7906933 33188621

[bib37] Juratli TA, McCabe D, Nayyar N, Williams EA, Silverman IM, Tummala SS, Fink AL, Baig A, Martinez-Lage M, Selig MK, (2018) DMD genomic deletions characterize a subset of progressive/higher-grade meningiomas with poor outcome. Acta Neuropathol 136: 779–792. 10.1007/s00401-018-1899-7 30123936

[bib38] Klinge L, Laval S, Keers S, Haldane F, Straub V, Barresi R, Bushby K (2007) From T-tubule to sarcolemma: Damage-induced dysferlin translocation in early myogenesis. FASEB J 21: 1768–1776. 10.1096/fj.06-7659com 17363620

[bib39] Korner H, Epanchintsev A, Berking C, Schuler-Thurner B, Speicher MR, Menssen A, Hermeking H (2007) Digital karyotyping reveals frequent inactivation of the dystrophin/DMD gene in malignant melanoma. Cell Cycle 6: 189–198. 10.4161/cc.6.2.3733 17314512

[bib40] Laemmli UK (1970) Cleavage of structural proteins during the assembly of the head of bacteriophage T4. Nature 227: 680–685. 10.1038/227680a0 5432063

[bib41] Levine MS, Bakker B, Boeckx B, Moyett J, Lu J, Vitre B, Spierings DC, Lansdorp PM, Cleveland DW, Lambrechts D, (2017) Centrosome amplification is sufficient to promote spontaneous tumorigenesis in mammals. Dev Cell 40: 313–322.e5. 10.1016/j.devcel.2016.12.022 28132847 PMC5296221

[bib42] Li Y, Huang J, Zhao YL, He J, Wang W, Davies KE, Nose V, Xiao S (2007) UTRN on chromosome 6q24 is mutated in multiple tumors. Oncogene 26: 6220–6228. 10.1038/sj.onc.1210432 17384672

[bib43] Liu J, Aoki M, Illa I, Wu C, Fardeau M, Angelini C, Serrano C, Urtizberea JA, Hentati F, Hamida MB, (1998) Dysferlin, a novel skeletal muscle gene, is mutated in Miyoshi myopathy and limb girdle muscular dystrophy. Nat Genet 20: 31–36. 10.1038/1682 9731526

[bib44] Luce LN, Abbate M, Cotignola J, Giliberto F (2017) Non-myogenic tumors display altered expression of dystrophin (DMD) and a high frequency of genetic alterations. Oncotarget 8: 145–155. 10.18632/oncotarget.10426 27391342 PMC5352069

[bib45] Luxton GWG, Gomes ER, Folker ES, Worman HJ, Gundersen GG (2011) TAN lines: A novel nuclear envelope structure involved in nuclear positioning. Nucleus 2: 173–181. 10.4161/nucl.2.3.16243 21818410 PMC3149877

[bib46] Luxton GWG, Gundersen GG (2011) Orientation and function of the nuclear-centrosomal axis during cell migration. Curr Opin Cell Biol 23: 579–588. 10.1016/j.ceb.2011.08.001 21885270 PMC3215267

[bib47] Manders EMM, Verbeek FJ, Aten JA (1993) Measurement of co-localization of objects in dual-colour confocal images. J Microsc 169: 375–382. 10.1111/j.1365-2818.1993.tb03313.x 33930978

[bib48] Matsumura K, Ervasti JM, Ohlendieck K, Kahl SD, Campbell KP (1992) Association of dystrophin-related protein with dystrophin-associated proteins in mdx mouse muscle. Nature 360: 588–591. 10.1038/360588a0 1461282

[bib49] Mauduit O, Delcroix V, Lesluyes T, Perot G, Lagarde P, Lartigue L, Blay JY, Chibon F (2019) Recurrent DMD deletions highlight specific role of Dp71 isoform in soft-tissue sarcomas. Cancers 11: 922. 10.3390/cancers11070922 PMC6678178 31266185

[bib50] Merle C, Thebault N, LeGuellec S, Baud J, Perot G, Lesluyes T, Delespaul L, Lartigue L, Chibon F (2020) Tetraploidization of immortalized myoblasts induced by cell fusion drives myogenic sarcoma development with DMD deletion. Cancers (Basel) 12: 1281. 10.3390/cancers12051281 PMC7281535 32438562

[bib51] Moretti D, Del Bello B, Allavena G, Corti A, Signorini C, Maellaro E (2015) Calpain-3 impairs cell proliferation and stimulates oxidative stress-mediated cell death in melanoma cells. PLoS One 10: e0117258. 10.1371/journal.pone.0117258 25658320 PMC4319969

[bib52] Moretti D, Del Bello B, Cosci E, Biagioli M, Miracco C, Maellaro E (2009) Novel variants of muscle calpain 3 identified in human melanoma cells: Cisplatin-induced changes in vitro and differential expression in melanocytic lesions. Carcinogenesis 30: 960–967. 10.1093/carcin/bgp098 19386580

[bib53] Muntoni F, Torelli S, Ferlini A (2003) Dystrophin and mutations: One gene, several proteins, multiple phenotypes. Lancet Neurol 2: 731–740. 10.1016/s1474-4422(03)00585-4 14636778

[bib54] Nigg EA (2002) Centrosome aberrations: Cause or consequence of cancer progression? Nat Rev Cancer 2: 815–825. 10.1038/nrc924 12415252

[bib55] Palazzo AF, Joseph HL, Chen YJ, Dujardin DL, Alberts AS, Pfister KK, Vallee RB, Gundersen GG (2001) Cdc42, dynein, and dynactin regulate MTOC reorientation independent of Rho-regulated microtubule stabilization. Curr Biol 11: 1536–1541. 10.1016/s0960-9822(01)00475-4 11591323

[bib56] Pihan GA (2013) Centrosome dysfunction contributes to chromosome instability, chromoanagenesis, and genome reprograming in cancer. Front Oncol 3: 277. 10.3389/fonc.2013.00277 24282781 PMC3824400

[bib57] Po’uha ST, Kavallaris M (2015) Gamma-actin is involved in regulating centrosome function and mitotic progression in cancer cells. Cell Cycle 14: 3908–3919. 10.1080/15384101.2015.1120920 26697841 PMC4825712

[bib58] Prins KW, Humston JL, Mehta A, Tate V, Ralston E, Ervasti JM (2009) Dystrophin is a microtubule-associated protein. J Cell Biol 186: 363–369. 10.1083/jcb.200905048 19651889 PMC2728405

[bib59] Raff JW, Basto R (2017) Centrosome amplification and cancer: A question of sufficiency. Dev Cell 40: 217–218. 10.1016/j.devcel.2017.01.009 28171744

[bib60] Reiss J, Rininsland F (1994) An explanation for the constitutive exon 9 cassette splicing of the DMD gene. Hum Mol Genet 3: 295–298. 10.1093/hmg/3.2.295 8004097

[bib61] Richard I, Broux O, Allamand V, Fougerousse F, Chiannilkulchai N, Bourg N, Brenguier L, Devaud C, Pasturaud P, Roudaut C, (1995) Mutations in the proteolytic enzyme calpain 3 cause limb-girdle muscular dystrophy type 2A. Cell 81: 27–40. 10.1016/0092-8674(95)90368-2 7720071

[bib62] Richard I, Roudaut C, Marchand S, Baghdiguian S, Herasse M, Stockholm D, Ono Y, Suel L, Bourg N, Sorimachi H, (2000) Loss of calpain 3 proteolytic activity leads to muscular dystrophy and to apoptosis-associated iκbα/nuclear factor κb pathway perturbation in mice. J Cell Biol 151: 1583–1590. 10.1083/jcb.151.7.1583 11134085 PMC2150676

[bib63] Roberds SL, Leturcq F, Allamand V, Piccolo F, Jeanpierre M, Anderson RD, Lim LE, Lee JC, Tome FM, Romero NB, (1994) Missense mutations in the adhalin gene linked to autosomal recessive muscular dystrophy. Cell 78: 625–633. 10.1016/0092-8674(94)90527-4 8069911

[bib64] Rodriguez OC, Schaefer AW, Mandato CA, Forscher P, Bement WM, Waterman-Storer CM (2003) Conserved microtubule-actin interactions in cell movement and morphogenesis. Nat Cell Biol 5: 599–609. 10.1038/ncb0703-599 12833063

[bib65] Schmidt WM, Uddin MH, Dysek S, Moser-Thier K, Pirker C, Höger H, Ambros IM, Ambros PF, Berger W, Bittner RE (2011) DNA damage, somatic aneuploidy, and malignant sarcoma susceptibility in muscular dystrophies. PLoS Genet 7: e1002042. 10.1371/journal.pgen.1002042 21533183 PMC3077392

[bib66] Sher RB, Cox GA, Mills KD, Sundberg JP (2011) Rhabdomyosarcomas in aging A/J mice. PLoS One 6: e23498. 10.1371/journal.pone.0023498 21853140 PMC3154500

[bib67] Tang H, Wei P, Chang P, Li Y, Yan D, Liu C, Hassan M, Li D (2017) Genetic polymorphisms associated with pancreatic cancer survival: A genome-wide association study. Int J Cancer 141: 678–686. 10.1002/ijc.30762 28470677 PMC5851439

[bib68] Vita GL, Politano L, Berardinelli A, Vita G (2021) Have duchenne muscular dystrophy patients an increased cancer risk? J Neuromuscul Dis 8: 1063–1067. 10.3233/jnd-210676 34024777

[bib69] Wang Y, Marino-Enriquez A, Bennett RR, Zhu M, Shen Y, Eilers G, Lee JC, Henze J, Fletcher BS, Gu Z, (2014) Dystrophin is a tumor suppressor in human cancers with myogenic programs. Nat Genet 46: 601–606. 10.1038/ng.2974 24793134 PMC4225780

[bib70] Wang YX, Feige P, Brun CE, Hekmatnejad B, Dumont NA, Renaud JM, Faulkes S, Guindon DE, Rudnicki MA (2019) EGFR-aurka signaling rescues polarity and regeneration defects in dystrophin-deficient muscle stem cells by increasing asymmetric divisions. Cell Stem Cell 24: 419–432.e6. 10.1016/j.stem.2019.01.002 30713094 PMC6408300

[bib71] Winter L, Abrahamsberg C, Wiche G (2008) Plectin isoform 1b mediates mitochondrion-intermediate filament network linkage and controls organelle shape. J Cell Biol 181: 903–911. 10.1083/jcb.200710151 18541706 PMC2426950

[bib72] Winter L, Staszewska I, Mihailovska E, Fischer I, Goldmann WH, Schröder R, Wiche G (2014) Chemical chaperone ameliorates pathological protein aggregation in plectin-deficient muscle. J Clin Invest 124: 1144–1157. 10.1172/jci71919 24487589 PMC3934181

[bib73] Zhou S, Ouyang W, Zhang X, Liao L, Pi X, Yang R, Mei B, Xu H, Xiang S, Li J (2021) UTRN inhibits melanoma growth by suppressing p38 and JNK/c-Jun signaling pathways. Cancer Cell Int 21: 88. 10.1186/s12935-021-01768-4 33632212 PMC7905598

