# Supplementary material for: Proteins implicated in muscular dystrophy and cancer are functional constituents of the centrosome
Source: Life Sci Alliance. 2022 Jul 5;5(11):e202201367. doi: 10.26508/lsa.202201367 (PMC9259872; doi:10.26508/lsa.202201367)

Full unedited gel for Figure 1E

Dystrophin:

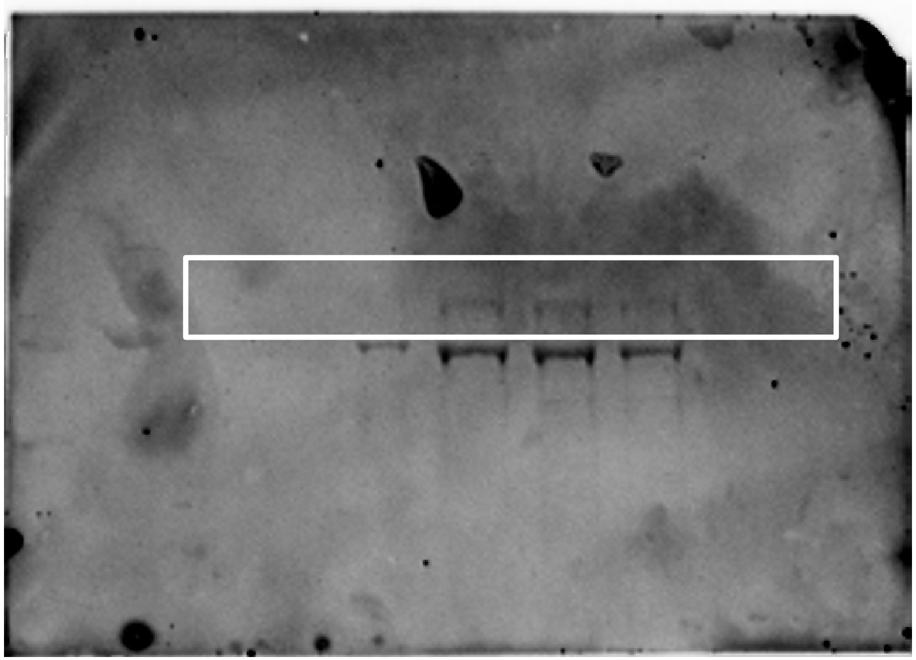

γ-Tubulin:

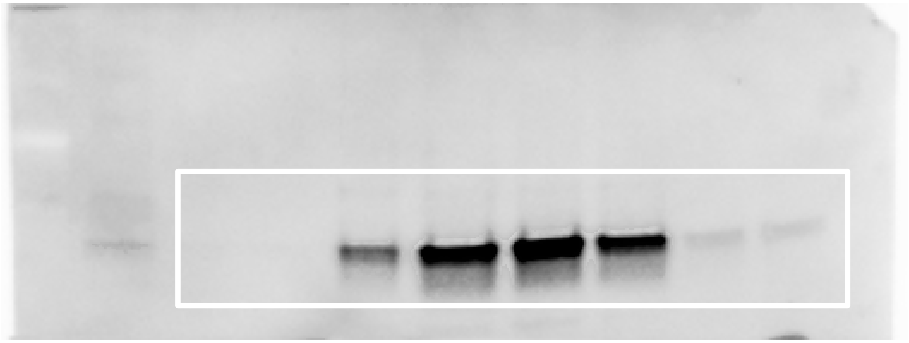

Full unedited gel for Figure 1G

Dystrophin:

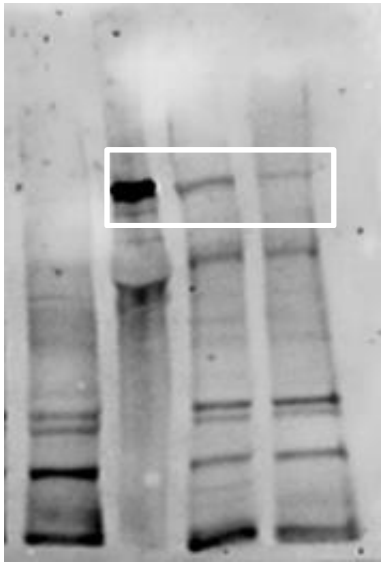

GAPDH:

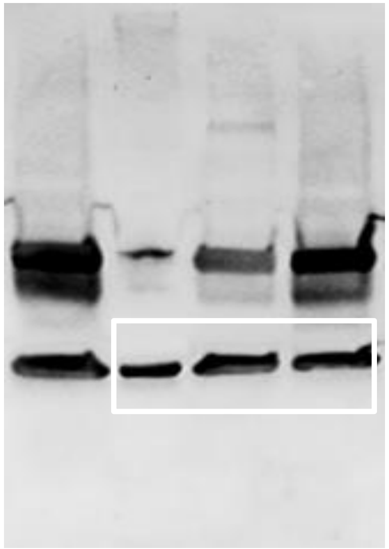

Full unedited gel for Figure 1H

Dystrophin:

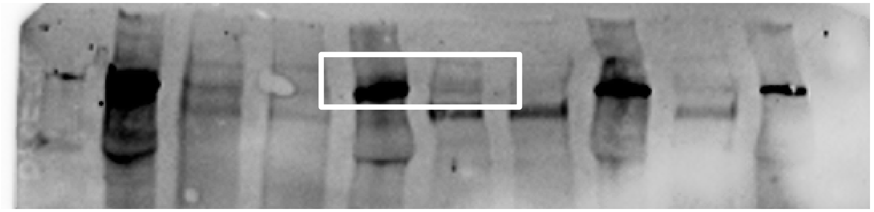

GAPDH:

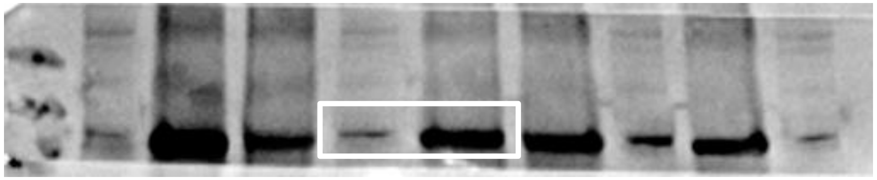

Full unedited gel for Figure 1J and 1K

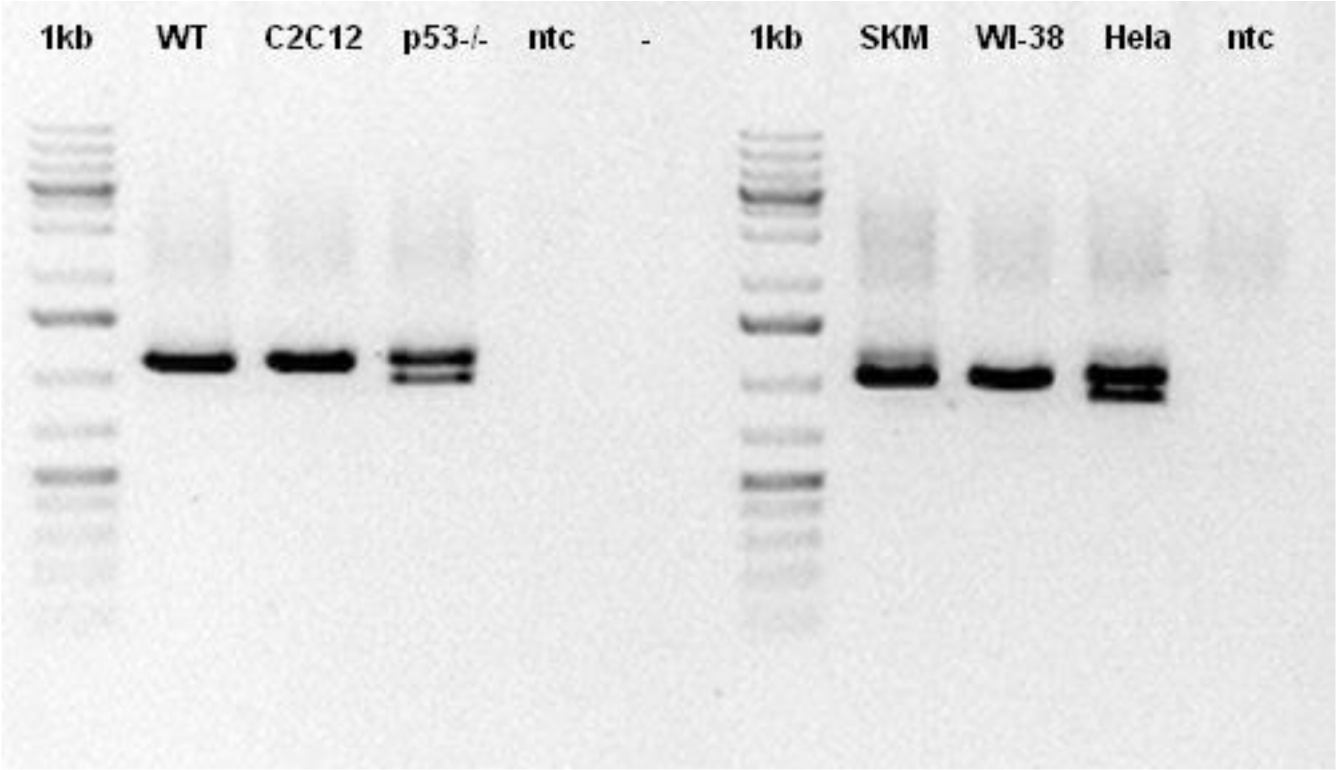

Full unedited gel for Figure 2E

Utrophin:

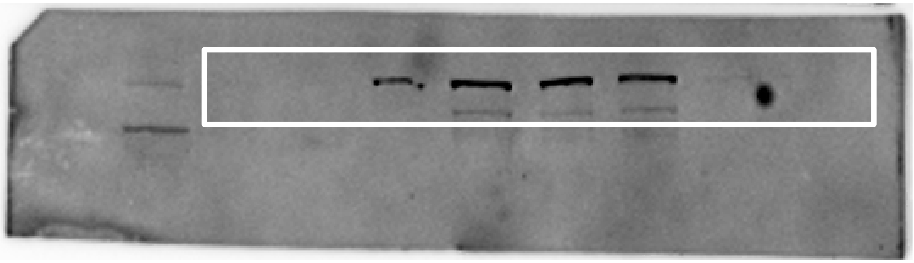

Dysferlin:

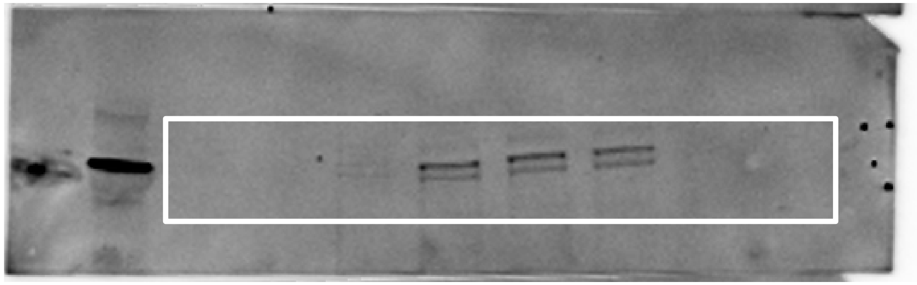

Calpain-3:

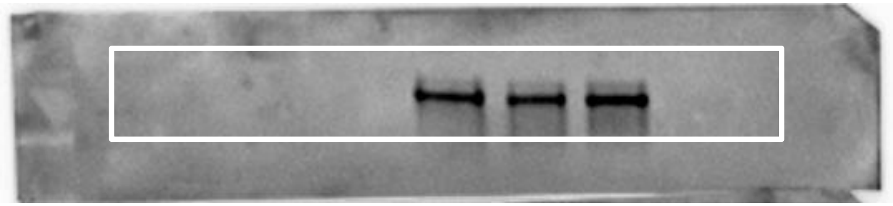

γ-Tubulin:

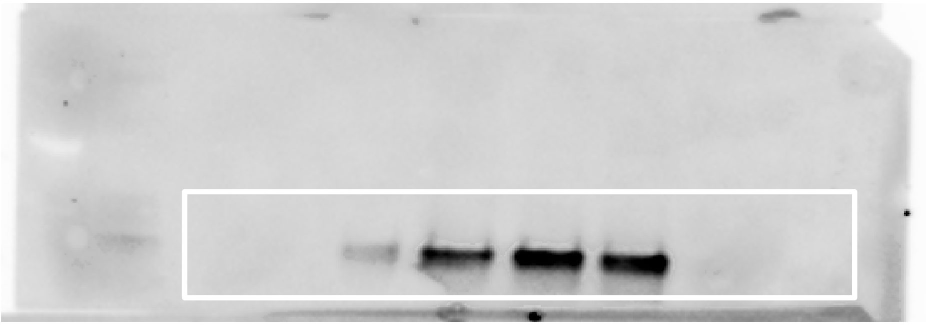

Full unedited gel for Figure 2G

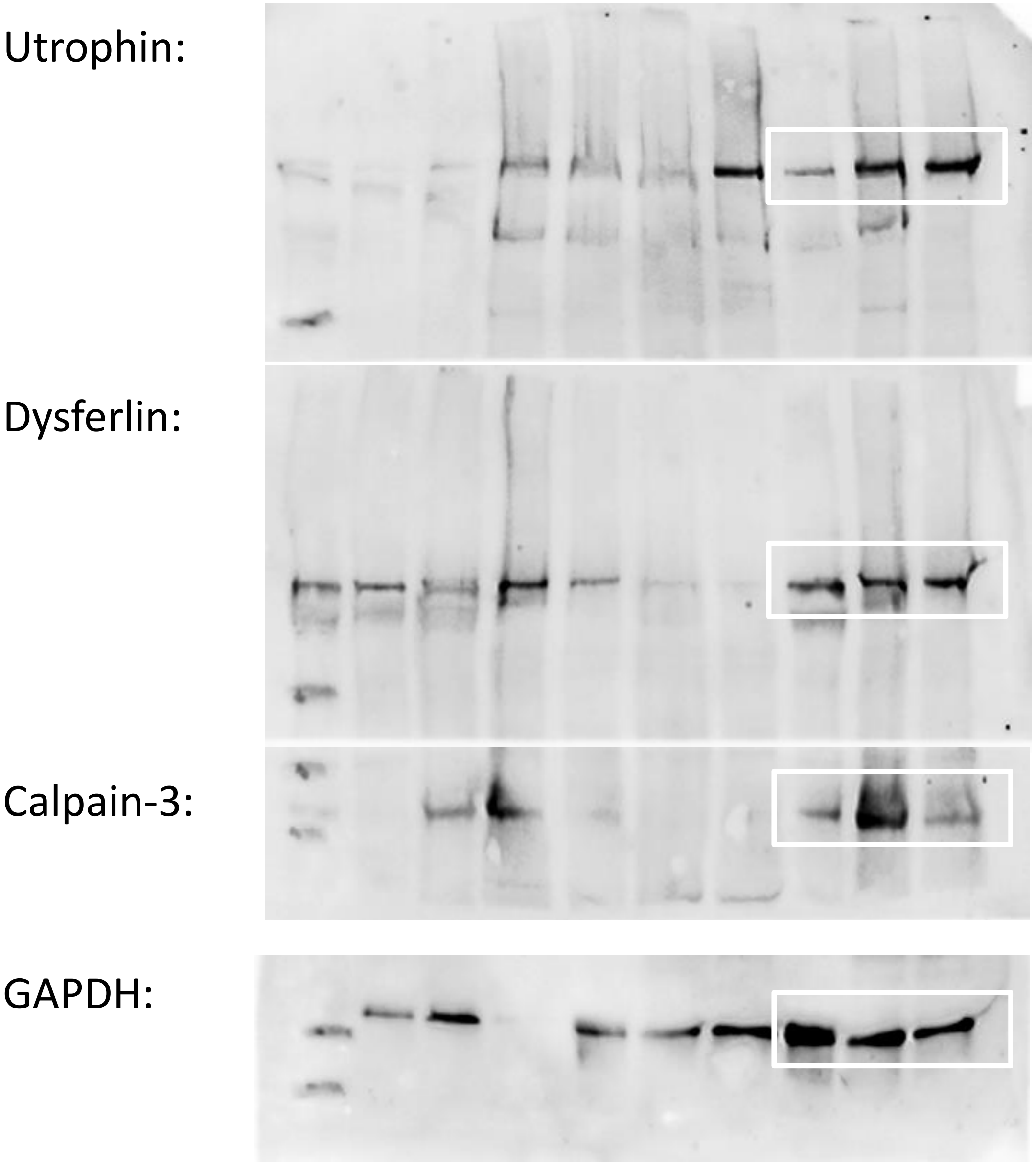

Full unedited gel for Figure 2H

Utrophin:

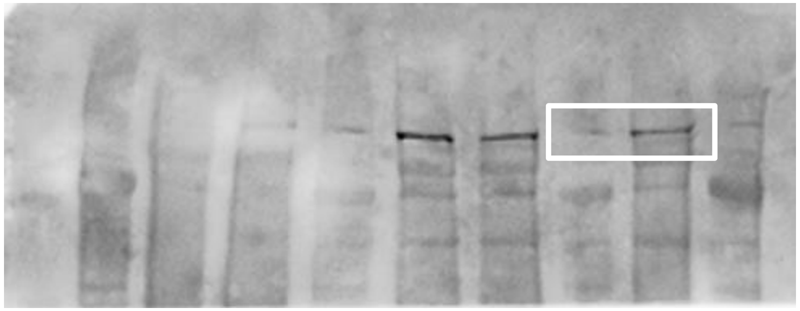

Dysferlin:

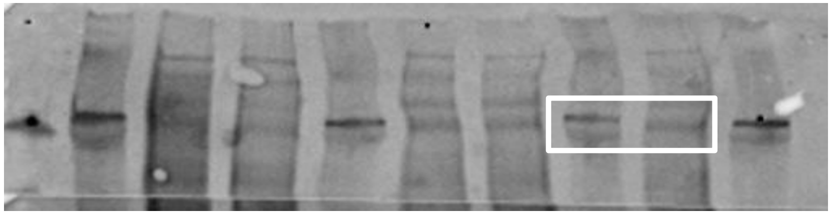

Calpain-3:

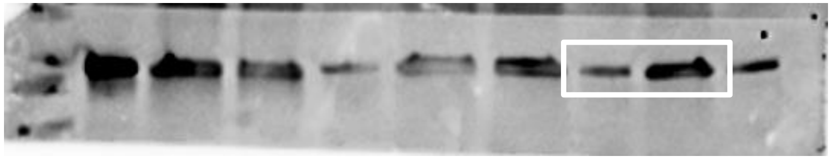

GAPDH:

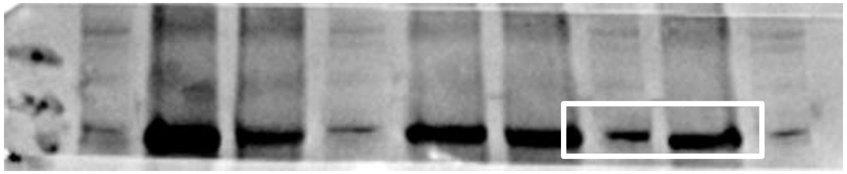

Same gel as for Figure 1H! (same experiment, but different lanes chosen for presentation)

Full unedited gel for Supplemental Figure 1A

Dystrophin:

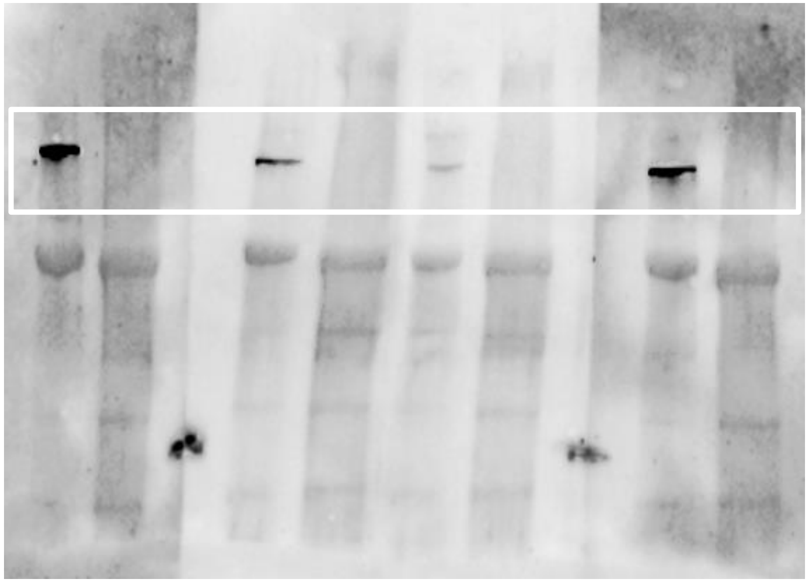

Full unedited gel for Supplemental Figure 3C

Dystrophin:

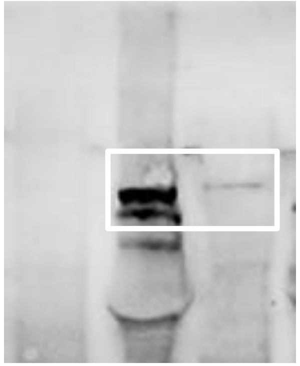

GAPDH:

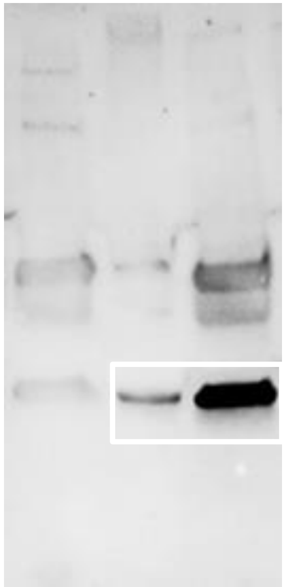

Supplement: Supplementary file 1 [file LSA-2022-01367_SdataF1_F2_FS1_FS3.pdf]
